# Transcatheter aortic valve implantation versus surgical aortic valve replacement for pure aortic regurgitation: a systematic review and meta-analysis of 33,484 patients

**DOI:** 10.1186/s12872-023-03667-0

**Published:** 2024-01-23

**Authors:** Mohamed Hamouda Elkasaby, Basma Badrawy Khalefa, Mazen Negmeldin Aly Yassin, Yasmeen Jamal Alabdallat, Ahmed Atia, Obieda Altobaishat, Islam Omar, Amany Hussein

**Affiliations:** 1 Faculty of Medicine, Al-Azhar University, Cairo, Egypt; 2 Faculty of Medicine, Ain Shams University, Cairo, Egypt; 3 Faculty of Medicine, Helwan University, Cairo, Egypt; 4 Faculty of Medicine, Hashemite University, Zarqa, Jordan; 5 Faculty of Medicine, Cairo University, Cairo, Egypt; 6 Faculty of Medicine, Jordan University of Science and Technology, Irbid, Jordan; 7 Faculty of Pharmacy, South Valley University, Qena, Egypt; 8 Medical Research Group of Egypt (MRGE), Cairo, Egypt

**Keywords:** Aortic regurgitation, Transcatheter aortic valve implantation, Surgical aortic valve replacement

## Abstract

**Introduction:**

The published studies comparing transcatheter aortic valve implantation (TAVI) and surgical aortic valve replacement (SAVR) in pure aortic regurgitation (AR) are conflicting. We conducted this systematic review and meta-analysis to compare TAVI with SAVR in pure AR.

**Methods:**

We searched PubMed, Embase, Web of Science (WOS), Scopus, and the Cochrane Library Central Register of Controlled Trials (CENTRAL) from inception until 23 June 2023. Review Manager was used for statistical analysis. The risk ratio (RR) with a 95% confidence interval (CI) was used to compare dichotomous outcomes. Continuous outcomes were compared using the mean difference (MD) and 95% CI. The inconsistency test (I^2^) assessed the heterogeneity. We used the Newcastle-Ottawa scale to assess the quality of included studies. We evaluated the strength of evidence using the Grading of Recommendations Assessment, Development, and Evaluation (GRADE) scale.

**Results:**

We included six studies with 5633 patients in the TAVI group and 27,851 in SAVR. In-hospital mortality was comparable between TAVI and SAVR (RR = 0.89, 95% CI [0.56, 1.42], *P* = 0.63) (I^2^ = 86%, *P* < 0.001). TAVI was favored over SAVR regarding in-hospital stroke (RR = 0.50; 95% CI [0.39, 0.66], *P* < 0.001) (I^2^ = 11%, *P* = 0.34), in-hospital acute kidney injury (RR = 0.56; 95% CI: [0.41, 0.76], *P* < 0.001) (*I*^*2*^ = 91%, *P* < 0.001), major bleeding (RR = 0.23; 95% CI: [0.17, 0.32], *P* < 0.001) (*I*^*2*^ = 78%, *P* < 0.001), and shorter hospital say (MD = − 4.76 days; 95% CI: [− 5.27, − 4.25], *P* < 0.001) (*I*^*2*^ = 88%, *P* < 0.001). In contrast, TAVI was associated with a higher rate of pacemaker implantation (RR = 1.68; 95% CI: [1.50, 1.88], *P* < 0.001) (*I*^*2*^ = 0% *P* = 0.83).

**Conclusion:**

TAVI reduces in-hospital stroke and is associated with better safety outcomes than SAVR in patients with pure AR.

**Supplementary Information:**

The online version contains supplementary material available at 10.1186/s12872-023-03667-0.

## Introduction

Aortic regurgitation (AR) is the third most common valvular disease in the population, and its prevalence is increasing owing to the aging population [1]. The prognosis for patients with AR, which can lead to severe left ventricular (LV) dysfunction, is dismal. *Dujardin* et al. reported a 10-year mortality rate of 34% in patients with severe AR who were conservatively treated [2]. Surgical aortic valve replacement (SAVR) remains the treatment of choice for severe AR, as referred to the American Heart Association (AHA), the American College of Cardiology (ACC) 2020 guidelines [3], the European Society of Cardiology (ESC), and the European Association for Cardio-Thoracic Surgery (EACTS) 2021 recommendations [4]. However, due to pre-existing comorbidities, some patients are often classified as high-risk and cannot undergo surgery, which necessitates a less invasive procedure [5].

In 2002, Cribier et al. performed the first transcatheter aortic valve implantation (TAVI) for patients with aortic valve stenosis (AS) who could not undergo SAVR [6]. Since then, there has been a significant increase in the number of TAVI procedures performed worldwide. A study reported a sixfold increase in procedure volume from 2012 to 2015 [7]. TAVI has been accepted by the US Food and Drug Administration (FDA) as a treatment for high-risk AS patients. Furthermore, the indications of TAVI have been expanded to include intermediate-risk patients in 2016 and low-risk patients in 2019 [8–11].

The expansion of TAVI to low-risk patients with AS, in addition to the exclusion of high-risk AR patients from surgery, has driven the exploration of TAVI as an alternative, off-label approach for non-surgical candidates with severe AR [12]. Several studies have demonstrated mainly favorable outcomes of TAVI in high-risk patients with AR, particularly among patients with newer-generation valves [13–16]. However, these studies are either case series or retrospective single-arm studies. Pivotal randomized controlled trials (RCTs) that investigated the safety and efficacy of TAVI in patients with AS excluded patients with pure native AR since AR can be associated with reduced valvular calcifications and annular dilatations, which leads to difficulties in anchoring the valves to their intended positions [17]. TAVI is generally well-defined for failing bioprosthetic tissue valves, which may fail due to aortic insufficiency. TAVI can also be used for patients with mixed valve disease [3]. However, the indications for TAVI in patients with pure native AR are not well-defined. The published studies comparing TAVI and SAVR in patients with AR are conflicting [18–21]. Prior meta-analyses have explored the feasibility of TAVI in patients with AR; however, these studies have not included a comparison with SAVR [22–24]. To date, no published meta-analysis has directly compared TAVI and SAVR in patients with pure AR.

To determine the best therapeutic option for patients with AR, we conducted a systematic review and meta-analysis to compare TAVI and SAVR in pure AR patients.

## Methods

We conducted a systematic review and meta-analysis, following the Cochrane Handbook for Systematic Reviews of Intervention [25] and reported it according to the Preferred Reporting Items for Systematic Reviews and Meta-Analyses (PRISMA) [26]. We also followed the AMSTAR-2 (Assessing the Methodological Quality of Systematic Reviews 2) guidelines [27]. Since this is a review study, patient consent and ethical approval were unnecessary. We registered the study protocol in PROSPERO (CRD42023431471).

### Search strategy

We searched PubMed, Embase, Web of Science (WOS), Scopus, and the Cochrane Library Central Register of Controlled Trials (CENTRAL) from inception until 23 June 2023. We utilized the keywords aortic regurgitation, TAVI, and SAVR. The search strategy for each database is illustrated in Supplementary Table 1.

### Inclusion criteria

We used the population, intervention, comparator, outcomes, and study design (PICOS) selection criteria to determine the included studies. We included studies with the following PICOS criteria. (1) Studies including patients with pure AR. (2) Intervention is TAVI. (3) Comparator is SAVR. (4) Studies included in-hospital mortality or stroke among the reported outcomes. (5) RCTs or cohort studies. We excluded single-arm studies, studies with more than one publication, studies including AS patients or patients with mixed AR and AS, case reports, reviews, abstracts, and animal studies.

The articles retrieved through the systematic search were uploaded to the EndNote Reference Library, where duplicates were determined and removed. After duplicates had been removed, the titles and abstracts of the search results were uploaded to the Rayyan website [28] and screened for relevance by two authors. Potentially eligible studies were then retrieved for full-text screening. The final list of included trials was agreed upon by discussion between all authors. Disagreement amongst reviewers was resolved through consensus. The reference lists of the retrieved studies were manually screened for any additional eligible studies.

### Data extraction

Extraction forms were constructed on Google Spread Sheets. Two authors extracted the data separately, and a third author solved disagreements. We extracted the following information for each study. (1) Summary of the included studies (the last name of the first author and the year of publication, study design, country, duration, sample size, details of each procedure, inclusion and exclusion criteria, implanted valve type, valve size, valve calcification, and duration of follow up). (2) Baseline characteristics including (Age, gender, body mass index (BMI), New York Heart Association classification of heart failure stage (NYHA), history of atrial fibrillation (AF), hypertension, diabetes, renal disease, liver disease, congestive heart failure, peripheral vascular disease, stroke or transient ischemic stroke, coronary artery disease, myocardial infarction (MI), previous percutaneous coronary intervention, and previous coronary artery bypass grafting surgery). (3) And outcomes including (mortality, major adverse composite cardiac events (MACCE), in-hospital stroke, MI, acute kidney injury (AKI), delirium, major bleeding, AF, pneumonia, sepsis, permanent pacemaker implantation (PPI), reintervention, and length of hospital stay (LOS)).

### Risk of bias assessment

The Newcastle Ottawa scale (NOS) for quality assessment of non-randomized trials [29] was used for the quality assessment of the included studies. The NOS assigns a maximum of nine points for the three domains: (1) Selection of study groups (four points); (2) Comparability of groups (two points); and 3) Ascertainment of exposure and outcomes (three points). NOS’s total score of 0 to 3 indicates a high risk of bias, 4 to 6 indicates a moderate risk, and ≥ 7 indicates a low risk of bias. Two independent authors performed the quality assessment separately. A third author resolved any disagreement.

### Data analysis

We used Review Manager (RevMan Version 5.4.1, The Cochrane Collaboration, 2020) for statistical analysis. To compare dichotomous outcomes, we used a risk ratio (RR) with a 95% confidence interval (CI) and the Mantel-Haenszel method; A *p*-value less than 0.05 was considered statistically significant. For continuous outcomes, we utilized the mean difference (MD) and 95% CI using the inverse variance method, and a *p*-value less than 0.05 was considered statistically significant. If we were comparing continuous outcomes with different measurement units, we used the standardized mean difference (SMD) and 95% CI instead of the MD; We only used this method when comparing the cost of procedures, as it was reported in US dollars and European Union euros. We used the inconsistency test (I^2^) to assess statistical heterogeneity and considered it significant when the I^2^ statistic exceeded 50% or had a *p*-value less than 0.10. We used the random effect model in our analysis. We meta-analyzed results for in-hospital, 30 days, and 1 year after the procedures. We did subgroup analysis depending on the approach of TAVI (transfemoral and transapical) and depending on the country of origin of the included studies. In the case of heterogeneity, we did a leave-one-out test by excluding one study in each scenario in order to eliminate the heterogeneity. We could not test for publication bias using Egger’s test since we did not include at least 10 studies for any of the outcomes, which is necessary to obtain accurate results [30].

### Assessment of the strength of the evidence

We evaluated the strength of evidence using the Grading of Recommendations Assessment, Development, and Evaluation (GRADE) scale. This scale assesses the certainty of the effect estimate. It has seven domains, including the number and design of studies, their quality, heterogeneity between pooled results, the direct effect of interventions, the precision of the confidence interval, and other considerations. GRADE categorizes evidence certainty into four levels: high quality, indicating that further research is unlikely to change the effects estimates; moderate quality, suggesting that further studies may affect confidence in effect estimation; low quality, indicating that additional research is crucial and likely to significantly impact the confidence of the effect estimation and may change the estimation; and very low quality, indicating uncertainty about the estimation.

## Results

### Search result

**Fig. 1:**
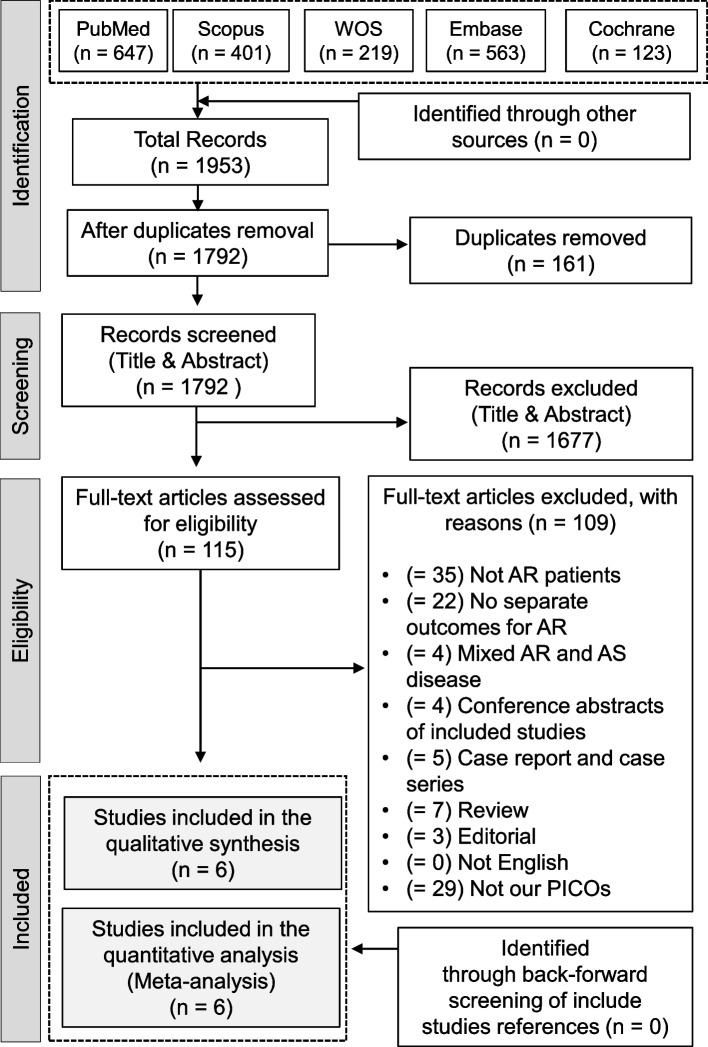
PRISMA flow diagram of the literature search results

Our search retrieved 1792 articles after removing the duplicates. Only 115 titles were eligible for full-text screening following the title and abstract screening. Finally, six studies were included [18–21, 31, 32] with a total of 5633 patients for TAVI and 27,851 patients for SAVR (PRISMA flow diagram; Fig. 1). All included studies were retrospective cohort studies, and the majority (three studies) were conducted in the United States, followed by Germany (two studies), and one study conducted in China. All included studies involved only patients with isolated AR. Table 1 summarizes the included studies and their most significant findings. Age in the TAVI group ranged from 77 ± 12.62 years to 67 ± 6.77 years, versus 75.6 ± 5.7 years to 60.9 ± 14.1 years in the SAVR group. The detailed baseline characteristics of patients in the included studies are illustrated in Table 2.

**Table 1:** Summary of the included studies

Study ID	Study design	Multicentric or single centric	Propensity-matched?	Country	Duration	Number of patients			Details of TAVI	Details of SAVR	Main inclusion criteria	Main exclusion criteria	Severity of AR	Implanted valve type	Valve size	Valve calcification	Follow-up period	Primary outcome
						TAVI	SAVR	Total										
Alharbi et al. 2020 [19]	Propensity-matched retrospective cohort study	Multicenter	Yes	United States	2016 to 2017	915	1390	2305	The NIS database lacks procedural details for TAVI and SAVR		Patients with pure AR	Infective endocarditis, concomitant AS, and those below the age of 18 years	NR	NR	NR	NR	In-hospital	In-hospital mortality
Mentias et al. 2023 [18]	Propensity-matched retrospective cohort study	Multicenter	Yes	United States	2016 to 2019	1147	9880	11,027	Transfemoral TAVI	NR	Patients with pure AR	Concomitant AS, valve-in-valve intervention, and other concomitant cardiac surgery or intervention	NR	NR	NR	NR	One year	One-year mortality
Oettinger et al. 2023 [31]	Retrospective cohort study	Multicenter	No	Germany	2018 to 2020	746	4025	4771	- Transapical TAVI - Transfemoral TAVI with a self-expandable and balloon-expandable valve	NR	Patients with pure AR	Concomitant AS, concomitant cardiac surgery or intervention	NR	Balloon-expandable and self-expanding valves	NR	NR	In-hospital	In-hospital mortality, major bleeding, and postoperative delirium
Rali et al. 2022 [32]	Retrospective cohort study	Multicenter	No	United States	2015 to 2018	105	50	155	The NIS database lacks procedural details for TAVR and SAVR.		Patients with pure AR	NR	NR	NR	NR	NR	NR	A composite of in-hospital mortality, stroke, transient ischemic stroke, myocardial infarction, pacemaker implantation, need for open surgery, vascular complications, and cardiac tamponade.
Stachon et al. 2020 [20]	Retrospective cohort study	Multicenter	No	Germany	2007 to 2015	724	10,528	11,252	Transapical and transfemoral TAVI	NR	Patients with pure AR and/or AS	NR	NR	NR	NR	NR	In-hospital	In-hospital mortality and complications (stroke, bleeding, post-operative delirium, ventilation, and length of hospital stay)
Zhou et al. 2023 [21]	Propensity-matched retrospective cohort study	Multicenter	Yes	China	2016 to 2019	1820	1820	3640	NR		Patients with AR	Concomitant AS, aged < 18 years, infective endocarditis, history of other cardiac surgeries	NR	NR	NR	NR	Six months	In-hospital mortality, mechanical ventilation, transfusion, sepsis, bacterial pneumonia, acute kidney injury, cardiac arrest, and intracranial hemorrhage

Abbreviations: *TAVI* transcatheter aortic valve implantation, *SAVR* surgical aortic valve replacement, *AR* aortic regurgitation, *ESC/EACTS* European Society of Cardiology/European Association for cardiothoracic surgery, *LVEF* left ventricular ejection fraction, *LVEED* left ventricular end-diastolic diameter, *NR* not reported, and *AS* aortic stenosis

**Table 2:** Baseline characteristics of patients in the included studies

Study ID	Study Groups	Sample	Age	BMI, Kg/m2	Sex, Female	NYHA classification		LVEF	LVEDD, cm	Comorbidities												
						III	IV			AF	Diabetes	HTN	COPD	Renal disease	Liver disease	CHF	PVD	History of MI	History of stroke or TIA	History of CAD	Previous CABG	Previous PCI
Alharbi et al. 2020 [19]	**TAVI**	915	77 (12.62)	–	265 (29.0)	–	–	–	–	–	115 (12.6)	410 (44.8)	230 (25.1)	365 (39.9)	35 (3.8)	730 (79.8)	200 (21.9)	–	–	575 (62.8)	–	–
	**SAVR**	1390	73.67 (9.65)	–	400 (28.8)	–	–	–	–	–	160 (11.5)	560 (40.3)	320 (23.0)	465 (33.5)	55 (4.0)	1060 (76.3)	350 (25.2)	–	–	830 (59.7)	–	–
Mentias et al. 2023 [18]	**TAVI**	1147	75.6 (6.8)	–	459 (40)	–	–	–	–	321 (28)	310 (27)	975 (85)	–	206 (18)	46 (4)	734 (64)	298 (26)	–	92 (8)	688 (60)	–	–
	**SAVR**	9880	75.6 (5.7)	–	3952 (40)	–	–	–	–	2766 (28)	2668 (27)	8483 (85)	–	1796 (18)	395 (4)	6323 (64)	2569 (26)	–	790 (8)	5928 (60)	–	–
Oettinger et al. 2023 [31]	**TAVI**	836	76.79 (8.77)	–	288 (34.45)	432 (51.67)		–	–	394 (47.12)	156 (18.66)	571 (68.3)	157 (18.78)	10 (1.2)	–	–	69 (8.25)	8 (0.96)	–	386 (46.17)	188 (22.49)	
	**SAVR**	4025	62.75 (13.58)	–	1023 (25.42)	1361 (33.81)		–	–	1790 (44.47)	491 (12.2)	2350 (58.4)	288 (7.16)	69 (1.71)	–	–	114 (2.83)	23 (0.57)	–	588 (14.61)	61 (1.52)	–
Rali et al. 2022 [32]	**TAVI**	105	67 (6.77)	–	40 (38.1)	–	–	–	–	35 (33.3)	35 (33.3)	85 (81)	20 (19)	30 (28.6)	–	–	–	–	–	55 (52.4)	–	–
	**SAVR**	50	61.33 (6.11)	–	15 (30)	–	–	–	–	20 (40)	NR	45 (90)	NR	30 (60)	–	–	–	–	–	30 (60)	–	–
Stachon et al. 2019 [20]	**TAVI**	724	77.04 (8.99)	–	298 (41.16)	350 (48.34)		–	–	319 (44.06)	150 (20.72)	455 (62.85)	109 (15.06)	16 (2.21)	–	–	77 (10.64)	12 (1.66)	–	317 (43.78)	161 (22.24)	–
	**SAVR**	10,528	60.9 (14.1)	–	2737 (26)	2653 (25.2)		–	–	3758 (35.7)	1284 (12.2)	5980 (56.8)	832 (7.9)	147 (1.4)	–	–	316 (3)	53 (0.5)	–	1063 (10.1)	253 (2.4)	–
Zhou et al. 2023 [21]	**TAVI**	1820	70.49 (12.49)	–	655 (36)	–	–	–	–	–	287 (15.8)	–	486 (26.7)	509 (28)	95 (5.2)	1334 (73.3)	465 (25.5)	232 (12.7)	213 (11.7)	–	202 (11.1)	206 (11.3)
	**SAVR**	1820	69.98 (10.41)	–	650 (35.7)	–	–	–	–	–	282 (15.5)	–	489 (26.9)	505 (27.7)	96 (5.3)	1326 (72.9)	439 (24.1)	237 (13)	211 (11.6)	–	213 (11.7)	212 (11.6)

Abbreviations: *ID* identification, *BMI* body mass index, *NYHA* New York Heart Association classification for heart failure severity, *LVEF* left ventricular ejection fraction, *LVEDD* left ventricular end-diastolic diameter, *AF* atrial fibrillation, *HTN* hypertension, *COPD* chronic obstructive pulmonary disease, *CHF* congestive heart failure, *PVD* peripheral vascular disease, *MI* myocardial infarction, *TIA* transient ischemic stroke, *CAD* coronary artery disease, *CABG* coronary artery bypass grafting, *PCI* percutaneous coronary intervention

### Quality of included studies

NOS determined that all included studies posed a low risk of bias. Table 3 displays the detailed quality assessment domains of the included studies.

**Table 3:** Risk of bias assessment of the included studies according to the Newcastle-Ottawa Quality Assessment Scale

Study ID	Alharbi et al. 2020 [19]	Mentias et al. 2023 [18]	Oettinger et al. 2023 [31]	Rali et al. 2022 [32]	Stachon et al. 2020 [20]	Zhou et al. 2023 [21]
Sample selection criteria (****)	****	****	****	****	****	****
**1) Representativeness of the exposed cohort** (a) Truly representative (one star) (b) Somewhat representative (one star) (c) Selected group (d) No description of the derivation of the cohort	a	a	a	a	a	a
**2) Selection of the non-exposed cohort** (a) Drawn from the same community as the exposed cohort (one star) (b) Drawn from a different source (c) No description of the derivation of the non-exposed cohort	a	a	a	a	a	a
**3) Ascertainment of exposure** (a) Secure record (e.g., surgical record) (one star) (b) Structured interview (one star) (c) Written self-report (d) No description (e) Other	a	a	a	a	a	a
**4) Demonstration that outcome of interest was not present at the start of the study** (a) Yes (one star) (b) No	a	a	a	a	a	a
Comparability (**)	*	**				**
**1) Comparability of cohorts on the basis of the design or analysis controlled for confounders** (a) The study controls for age (one star) (b) Cohorts are not comparable on the basis of the design or analysis controlled for age (c) No available separate baseline data for the included population in the current meta-analysis	b	a	b	b	b	a
(a)Study controls for other comorbidities^$^ (one star) (b) Cohorts are not comparable on the basis of the design or analysis controlled for comorbidities (c) No available separate baseline data for the included population in the current meta-analysis	a	a	b	b	b	a
Exposure (***)	***	***	***	***	***	***
**1) Assessment of outcome** (a) Independent blind assessment (one star) (b) Record linkage (one star) (c) Self-report (d) No description (e) Other	a	a	a	a	a	a
**2) Was follow-up long enough for outcomes to occur** (a) Yes (one star) (b) No	a	a	a	a	a	a
**3) Adequacy of follow-up of cohorts** (a) Complete follow up- all subjects accounted for (one star) (b) Subjects lost to follow-up unlikely to introduce bias- number lost less than or equal to 20% or description of those lost suggested no different from those followed. (one star) (c) Follow up rate less than 80% and no description of those lost (d) No statement	a	a	a	a	a	a
**Summary risk of bias score**	**Low**	**Low**	**Low**	**Low**	**Low**	**Low**

(*) Emphasizes that this domain is of high quality and the risk of bias is minimal

### Primary safety outcomes

#### In-hospital mortality

**Fig. 2:**
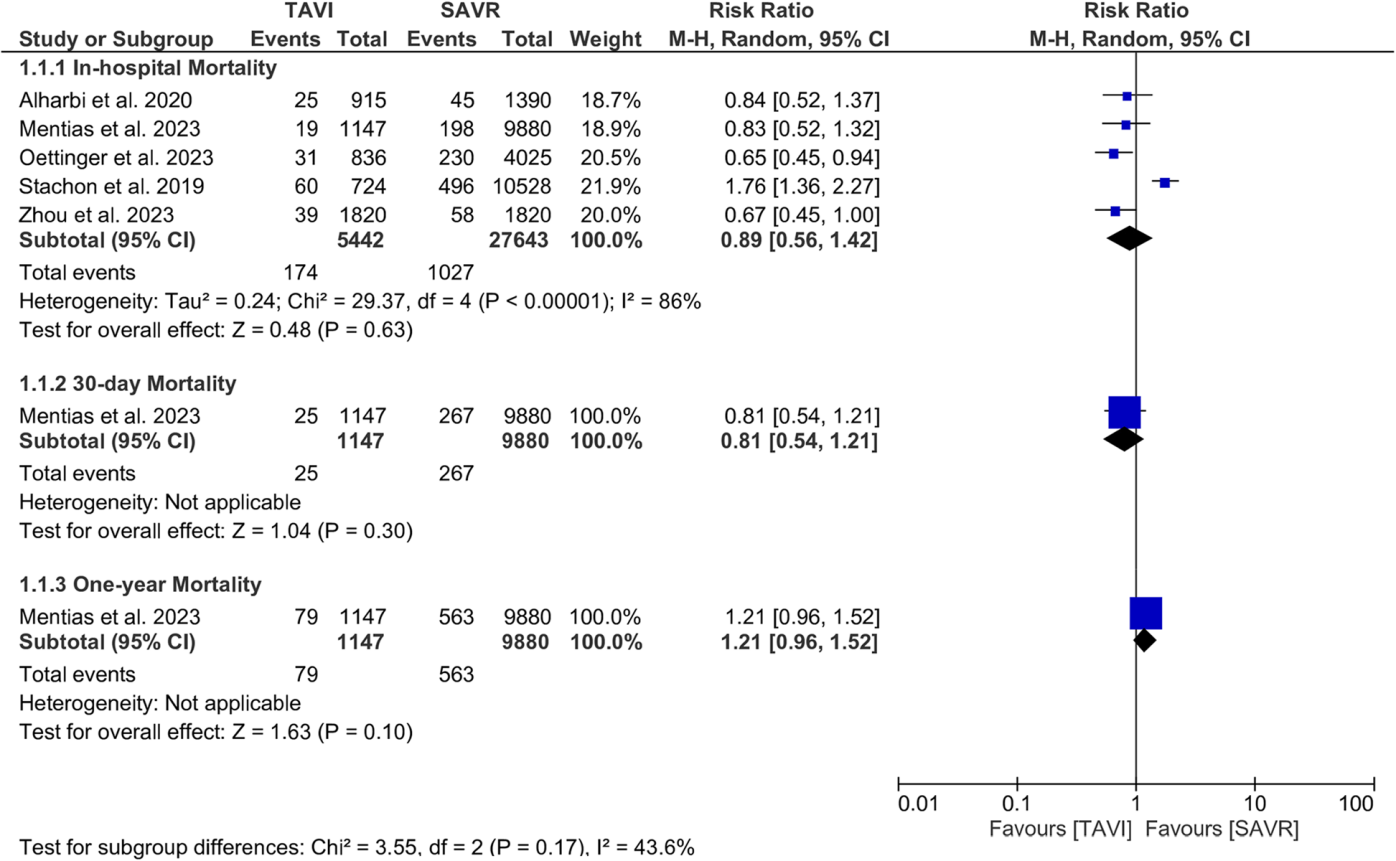
Forest plot of risk ratio (RR) and 95% confidence interval (CI) in the mortality; subtotals only. Abbreviations: TAVI; transcatheter aortic valve implantation, SAVR; aortic valve replacement, and M-H; Mantel-Haenszel method

In-hospital mortality was comparable between the two procedures (RR = 0.89, 95% CI [0.56, 1.42], *P* = 0.63); The pooled results were not homogenous (*I*^*2*^ = 86%, *P <* 0.001), Fig. 2. Heterogeneity was best addressed by excluding the study of Stachon et al. 2019 [20] (*I*^*2*^ = 0%, *P* = 0.84). After removing Stachon et al. 2019 [20] from the meta-analysis, the overall RR favored TAVI over SAVR (RR = 0.72; 95% CI: [0.59, 0.89], *P* = 0.003), Supplementary Fig. 1.

Transapical TAVI was associated with an increased in-hospital mortality rate compared to SAVR (RR = 1.53; 95% CI [1.02, 2.31], *P* = 0.04) (*I*^*2*^ = 0%, *P* = 0.47). Transfemoral TAVI was associated with a similar in-hospital mortality rate compared to SAVR (RR = 0.99; 95% CI [0.48, 2.04], *P* = 0.97) (*I*^*2*^ = 91%, *P* < 0.001). While pooled results of undefined TAVI approaches showed a lower rate of in-hospital mortality compared to SAVR (RR = 0.60; 95% CI [0.41, 0.87], *P* = 0.008) (*I*^*2*^ = 9%, *P* = 0.30), Supplementary Fig. 2.

TAVI was favored over SAVR in studies conducted in China (RR = 0.67; CI: [0.45, 0.1], *P* = 0.05). There were no differences between TAVI and SAVR in the USA (*P* = 0.29) and Germany (*P* = 0.88) subgroups, Supplementary Fig. 3.

#### 30-day and one-year mortality

Only one study [18] reported the mortality rates at 30 days and 1 year of follow-up. The results of this study did not favor either of the two procedures at 30-day follow-up (RR = 0.81; 95% CI: [0.54,1.21], *P* = 0.30) or one-year (RR = 1.21; 95% CI: [0.98,1.52], *P* = 0.1), Fig. 2.

#### Stroke

**Fig. 3:**
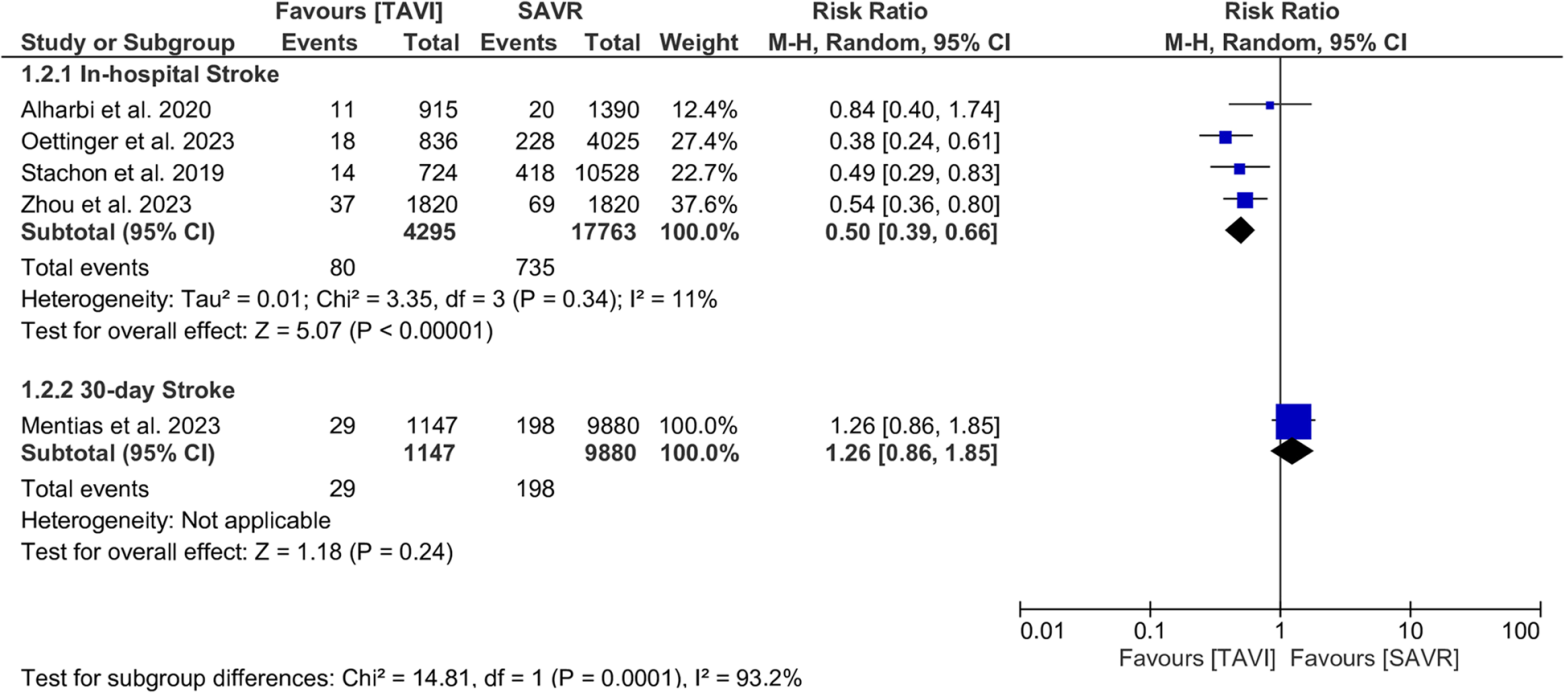
Forest plot of risk ratio (RR) and 95% confidence interval (CI) in the stroke; subtotals only. Abbreviations: TAVI; transcatheter aortic valve implantation, SAVR; aortic valve replacement, and M-H; Mantel-Haenszel method

In-hospital stroke was lower in TAVI than SAVR (RR = 0.50; 95% CI [0.39, 0.66], *P* <0.001), the pooled results were not significantly heterogenous (*I*^*2*^ = 11%, *P* = 0.34), Fig. 3.

We found that transapical TAVI was not protective against stroke compared to SAVR (RR = 0.64; 95% CI: [0.31, 1.35], *P* = 0.24) (*I*^*2*^ = 1%, *P* = 0.31), while transfemoral TAVI approach was protective compared to SAVR (RR = 0.39; 95% CI: [0.26, 0.59], *P* < 0.001) (*I*^*2*^ = 0%, *P* = 0.85). Also, the undefined TAVI approach was associated with a lower rate of in-hospital stroke (RR = 0.60; CI: [0.41, 0.87], *P* = 0.008) (*I*^*2*^ = 9%, *P* = 0.30), Supplementary Fig. 4.

There was no difference between TAVI and SAVR in the USA (RR = 0.84; CI: [0.40, 1.74], *P* = 0.63). While TAVI was protective in Germany (RR = 0.42; CI: [0.30, 0.60], *P* < 0.001) (*I*^*2*^ = 0%, *P* = 0.49) and China (RR = 0.54; 95%CI: [0.36, 0.80], *P* = 0.002), Supplementary Fig. 5.

30-day stroke was similar in TAVI and SAVR (RR = 1.26; 95% CI [0.86, 1.85], *P* = 0.24). This outcome was reported only in one study [18], Fig. 3.

#### Postoperative atrial fibrillation

**Fig. 4:**
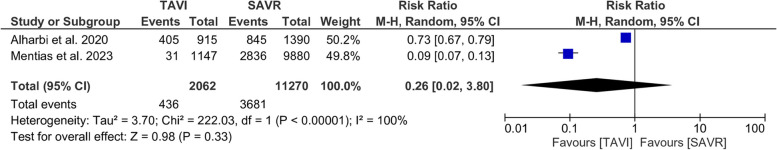
Forest plot of risk ratio (RR) and 95% confidence interval (CI) in the postoperative atrial fibrillation (AF). Abbreviations: TAVI; transcatheter aortic valve implantation, SAVR; aortic valve replacement, and M-H; Mantel-Haenszel method

The overall RR did not favor either of the two procedures regarding postoperative AF (RR = 0.26; 95% CI: [0.02, 3.80], *P* = 0.33). The pooled studies were not homogenous (*I*^*2*^ = 100%, *P*  <0.001), Fig. 4.

#### Acute kidney injury

**Fig. 5:**
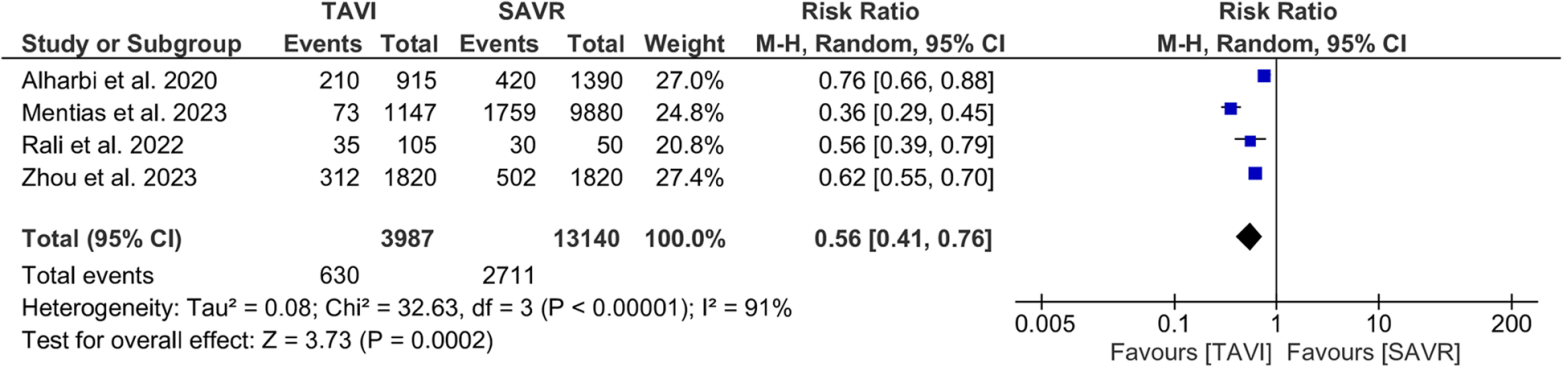
Forest plot of risk ratio (RR) and 95% confidence interval (CI) in the postoperative acute kidney injury (AKI), subtotals only. Abbreviations: TAVI; transcatheter aortic valve implantation, SAVR; aortic valve replacement, and M-H; Mantel-Haenszel method

In-hospital AKI was lower in TAVI than SAVR (RR = 0.56; 95% CI: [0.41, 0.76], *P* < 0.001). The pooled results were heterogeneous (*I*^*2*^ = 91%, *P* < 0.001), Fig. 5. A leave-one-out test could not address the source of heterogeneity.

The pooled result favored transfemoral TAVI over SAVR (RR = 0.36; 95% CI: [0.29, 0.45], *P* < 0.001), and the undefined approach over SAVR (RR = 0.66; 95% CI: [0.56, 0.78], *P* < 0.001) (*I*^*2*^ = 63%, *P = 0.07*), Supplementary Fig. 6.

#### Major bleeding

**Fig. 6:**
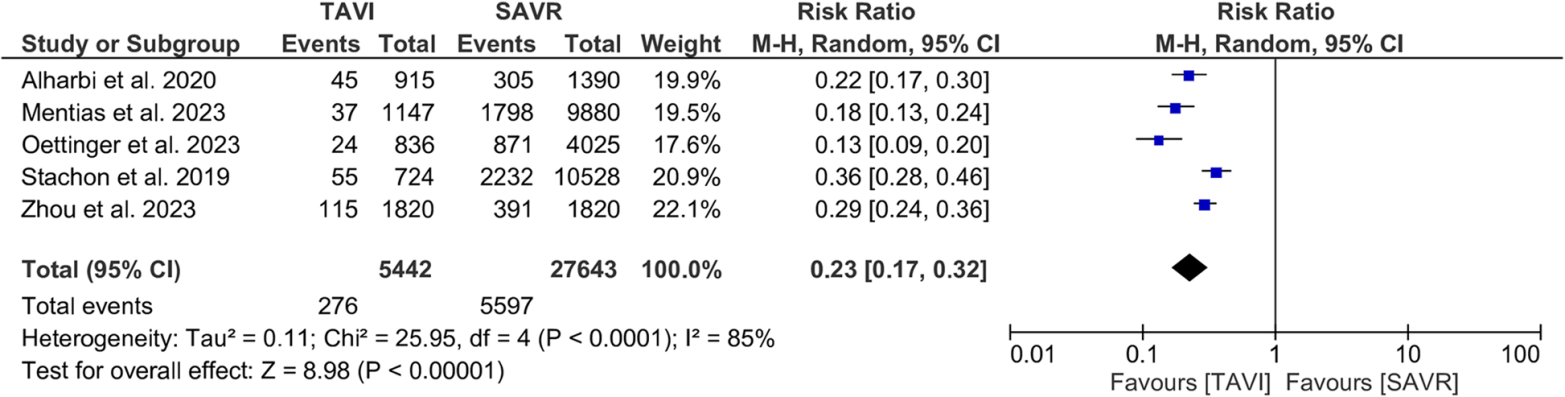
Forest plot of risk ratio (RR) and 95% confidence interval (CI) in the postoperative major bleeding. Abbreviations: TAVI; transcatheter aortic valve implantation, SAVR; aortic valve replacement, and M-H; Mantel-Haenszel method

TAVI was associated with a significantly lower risk of major bleeding than SAVR (RR 0.23, 95% CI [0.17, 0.32], *P*  <0.001). The pooled results were not homogenous (*I*^*2*^ = 85%, *P* < 0.001), Fig. 6. The leave-one-out test did not address the source of heterogeneity.

Transapical TAVI was favored over SAVR (RR = 0.41; 95% CI: [0.28, 0.59], *P* < 0.001), with homogenous results (*I*^*2*^ = 0%, *P* = 0.81). Transfemoral and undefined TAVI approaches both were favored over SAVR, but their pooled results were heterogenous (RR = 0.19; 95% CI: [0.11, 0.34], *P* < 0.001) (*I*^*2*^ = 87%, *P* < 0.001) and (RR = 0.26; 95% CI: [0.20, 0.34], *P* < 0.001) (*I*^*2*^ = 55%, *P* = 0.14) respectively, Supplementary Fig. 7.

### Secondary outcomes

#### Permanent pacemaker implantation

**Fig. 7:**
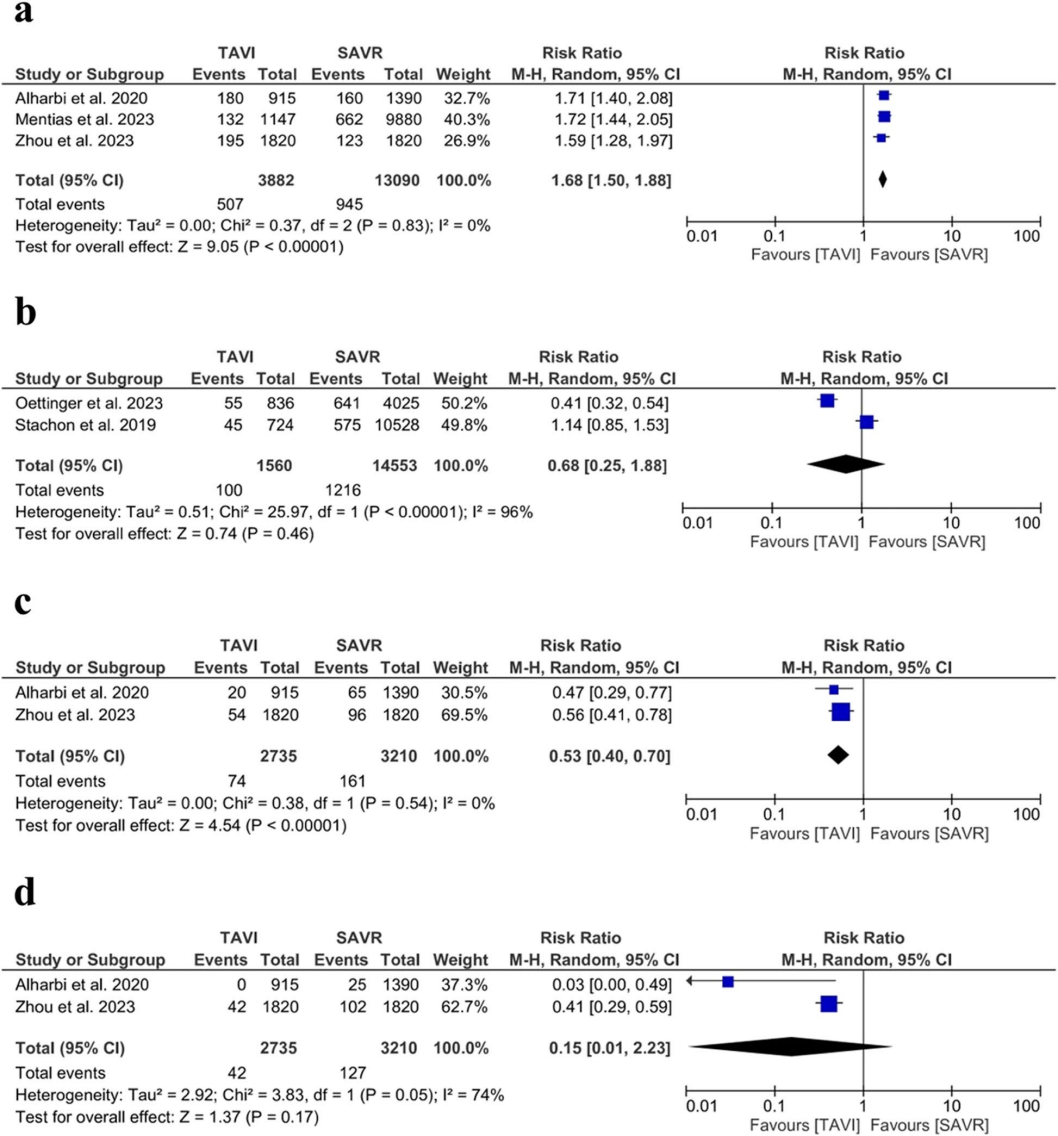
Forest plot of risk ratio (RR) and 95% confidence interval (CI) in the secondary safety outcomes. **a** Permanent pacemaker implantation (PPI). **b** Delirium. **c** Pneumonia. **d** Sepsis. Abbreviations: TAVI; transcatheter aortic valve implantation, SAVR; aortic valve replacement, and M-H; Mantel‐Haenszel method

TAVI was associated with a higher rate of PPI (RR = 1.68; 95% CI [1.50, 1.88], *P*  <0.001). The pooled studies were homogenous (*I*^*2*^ = 0% *P* = 0.83). Figure 7a.

#### Delirium

The overall RR between the TAVI and the SAVR did not favor either of the two procedures (RR = 0.68; 95% CI: [0.25, 1.88], *P* = 0.46). The pooled studies were not homogenous (*I*^*2*^ = 96%, *P* < 0.001). Due to the limited number of studies, it is impossible to address the heterogeneity through leave-one-out or subgroup analysis, Fig. 7b.

#### Pneumonia

SAVR was associated with an increased risk of pneumonia than TAVI (RR = 0.53; 95% CI: [0.40, 0.70], *P* < 0.001). The pooled studies were not homogenous (*I*^*2*^ = 0%, *P* = 0.54), Fig. 7c.

#### Sepsis

The overall effect estimate did not favor either of the two procedures (RR = 0.15; 95% CI: [0.01, 2.23], *P* = 0.17); The pooled studies were not homogenous (*I*^*2*^ = 74%, *P* = 0.05), Fig. 7d. Due to the limited number of studies, it is impossible to address the heterogeneity through leave-one-out or subgroup analysis.

#### Myocardial infarction

MI was reported only in one study [19], which showed no difference between TAVI and SAVR (RR = 0.79; 95% CI: [0.59, 1.05], *P* = 0.11).

#### Major Adverse Composite Cardiac Events

MACCE was reported only in one study [32], which favored TAVI (RR = 0.48; 95% CI: [0.25, 0.90], *P* = 0.02).

### Healthcare system utilization

#### Length of hospital stay

**Fig. 8:**
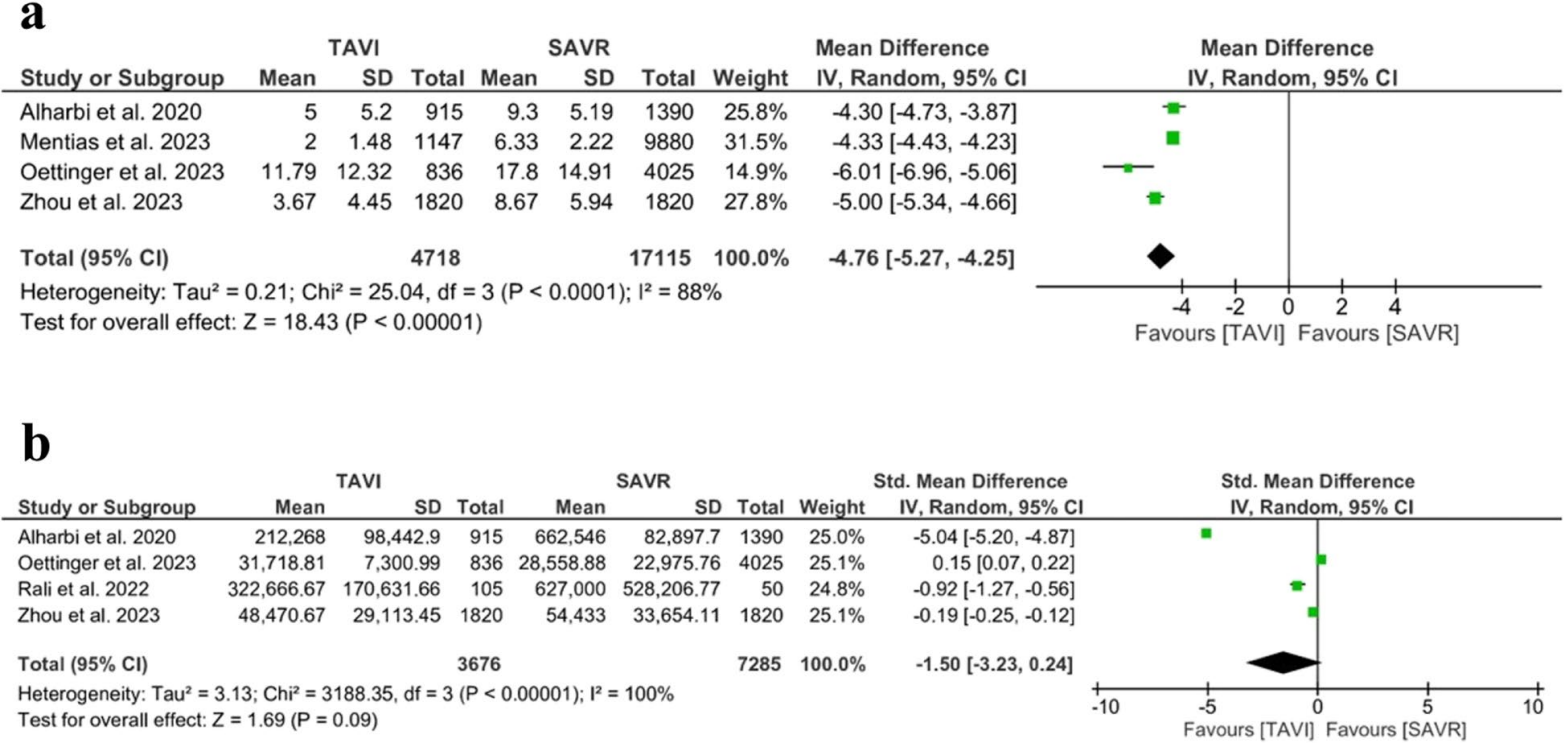
Forest plots of risk ratio (RR) and 95% confidence interval (CI) in the healthcare system utilization outcomes. **a** Length of hospital stay. **b** Cost. Abbreviations: TAVI; transcatheter aortic valve implantation, SAVR; aortic valve replacement, and I-V; inverse variance method

The overall effect estimate favored the TAVI regarding the LOS (MD = − 4.76 days; 95% CI: [− 5.27, − 4.25], *P* <0.001). The pooled studies were not homogenous (*I*^*2*^ = 88%, *P*  <0.001), Fig. 8a. The leave-one-out test did not address the source of heterogeneity.

Transfemoral TAVI was associated with shorter LOS compared to SAVR (MD = − 4.33 days, 95% CI: [− 4.42, − 4.23], *P* < 0.001), and the results were homogenous (*I*^*2*^ = 0%, *P* = 0.59). Transapical TAVI was not associated with decreased LOS compared to SAVR (MD = − 1.98 days, 95% CI: [− 4.33, 0.93], *P* = 0.21). The undefined TAVI approach subgroup was associated with shorter LOS compared to SAVR (MD = − 4.66 days, 95% CI: [− 5.35, − 3.98], *P* < 0.001) (*I*^*2*^ = 84%, *P* = 0.01), Supplementary Fig. 8.

#### Cost

The meta-analysis of cost did not favor either of the two groups (SMD = − 1.50; 95% CI: [− 3.23, 0.24], *P* = 0.09). The pooled studies were not homogenous (*I*^*2*^ = 100%, *P* > 0.001). We could not address the source of heterogeneity either by leave-one-out test or subgroup analysis, Fig. 8b.

### Strength of the evidence

The results of the strength of the evidence according to GRADE are summarized in Supplementary Table 2. The GRADE system classified the strength of evidence as moderate for in-hospital stroke. Low for in-hospital mortality, AKI, major bleeding, and PPI. And very low for AF, delirium, and sepsis.

## Discussion

### Summary of the key findings

The present study aimed to compare the safety and efficacy of TAVI and SAVR for the treatment of AR. The meta-analysis revealed important findings regarding mortality rates, procedural complications, and healthcare system utilization. The mortality rates between the two procedures were comparable. TAVI demonstrated advantages over SAVR in terms of stroke, major bleeding, AKI, pneumonia, and shorter LOS. However, TAVI was associated with a higher risk of PPI.

 Franzone et al. 2016 [23], Jiang et al. 2017 [22], and Takagi et al. 2020 [24] conducted single-arm meta-analysis studies of the feasibility of TAVI in AR patients. A small number of patients limited these studies and did not compare TAVI with SAVR. To the best of our knowledge, our study is the first to compare TAVI with SAVR in patients with pure AR.

The mortality rates between TAVI and SAVR were comparable. This finding aligns with previous research and supports the notion that TAVI is not inferior to SAVR regarding overall patient survival in AR [18, 19, 31] and also AS patients [33–35]. In our cohort, TAVI patients were older and had higher comorbidity scores, which aligns with the current recommendations and practice directions that TAVR is assigned only to patients with higher risk who cannot undergo surgery [3, 5]. This may be why TAVR was associated with a lower number of deaths but did not reach statistical significance. *Franzone* et al. 2016 [23], *Jiang* et al. 2017 [22], and *Takagi* et al. 2020 [23] reported a 30-day all-cause mortality after TAVI of 8%, 12, 9%, and 9.5, respectively. Our study found that the rate of all-cause in-hospital mortality after TAVI was 3.1%. Although our study had a shorter follow-up period (in-hospital) compared to previous single-arm meta-analysis studies, we believe that the mortality rate after TAVI is decreasing due to the growing experience of the operators. The most recent studies report a mortality rate after TAVI of 3.7 and 2.01% among patients treated between 2018 and 2020 and 2016–2019, respectively [21, 31]. The source of heterogeneity in our analysis was Stachon et al. 2020 [20]; after excluding it, the overall RR favored the TAVI over SAVR (RR = 0.72; 95% CI: [0.59, 0.89], *P* = 0.003). Stachon et al. 2020 [20] reported an increased risk of in-hospital mortality with TAVI compared to SAVR. The different results may be because it was an early study conducted in 2008, which may resemble a limited experience of operators and less advanced technology. The sensitivity analysis after excluding Stachon et al. 2020 [20] suggests that TAVI may be associated with a decreased mortality rate than SAVR.

The decreased risk of postoperative stroke to half the incidence after SAVR is a very interesting finding of this meta-analysis, which suggests the superiority of TAVI in AR patients. Our results align with previous reports [21, 31].

Although TAVI has made significant progress over the past 20 years with improved devices and cardiologist experience, stroke remains a major concern associated with this procedure with AS. It can increase mortality rates and decrease the patient’s quality of life. Different reports have shown that the incidence of stroke within 30 days post-TAVI ranges from 0.6 to 6.7% with AS [9–11, 36, 37]. Stroke after TAVI can have varying symptoms, from disabling or non-disabling stroke to silent stroke detected only by diffusion-weighted magnetic resonance imaging (DW-MRI) [38, 39]. 75% of AS patients experience debris embolization during TAVI of the calcified aortic arch and valve, posing a risk of stroke of any type [40].

Previous single-arm meta-analyses reported that stroke was an infrequent event in patients with AR who underwent TAVI. *Franzone* et al. 2016 [23] reported a 0% incidence of stroke, *Jiang* et al. 2017 [22] reported a 0.01% incidence of stroke, and *Takagi* et al. 2020 [24] reported a 2.9% incidence of stroke. However, these studies were limited by a small number of patients; our study also found similar findings, with only 1.8% of patients experiencing stroke in the TAVI group. AR is not always associated with calcification [41], so the decreased risk of stroke may be due to a different underlying pathology.

TAVI demonstrated advantages over SAVR, including bleeding, AKI, pneumonia, and shorter LOS, aligning with previous research [18, 19, 21, 32, 42, 43]. These benefits are consistent with the less invasive nature of TAVI, which avoids sternotomy and cardiopulmonary bypass. The shorter LOS associated with TAVI compared to SAVR suggests potential cost savings and improved resource allocation within healthcare systems. These findings can inform healthcare providers, policymakers, and administrators in making informed decisions regarding adopting and allocating resources for these interventions.

The increased risk of PPI after TAVI is not surprising, as shown in previous research on patients with AS and AR [18, 21, 35, 43, 44]. *Franzone* et al. 2016 [23] reported a PPI after a TAVI rate of 11%, *Jiang* et al. 2017 reported 11% [22], and *Takagi* et al. 2020 reported 11.6% [24]. The rate of PPI after TAVI in our study was near to the previous results (13.06%). It is assumed that injury to the superficial atrioventricular and left bundles during implantation is the direct cause of PPI after TAVI. Regardless of the condition, this injury is believed to be related to the difficulty of anchoring the valve. The superior anchoring mechanisms of the newer valves resulted in better PPI outcomes, but this improvement was not statistically significant [13]. This finding highlights the need for careful patient selection and diligent postoperative monitoring, particularly in patients at risk for conduction disturbances.

Healthcare providers need to assess the individual risk of each patient and involve them in the decision-making process. Factors such as age, comorbidities, anatomical considerations, and the surgeon’s expertise should be considered when determining the best procedure for a patient.

### Strengths and limitations

This study investigates the comparative efficacy of TAVI and SAVR in managing pure AR. The study’s strength lies in its clinical relevance, as it addresses a pressing issue in cardiology. It directly compares TAVI and SAVR in a meta-analysis for the first time, including six studies and a large number of pure AR patients. The study aimed to provide valuable insights into each intervention’s relative benefits and risks. This study investigated a large scope of clinical outcomes. Also, it provided a valuable subgroup analysis to deepen our understanding of each TAVI approach separately and if the results differ from one country to another. We were able to address the source of heterogeneity in many outcomes. The findings can potentially guide clinical decision-making and improve patient care by informing physicians about the most appropriate treatment option.

Considering the economic implications of TAVI and SAVR on healthcare utilization further enhances the study’s impact. Ultimately, this research fills a knowledge gap and advances our understanding of aortic valve disease management, making it valuable for clinicians and policymakers.

While the study has several strengths, it is also important to acknowledge its weaknesses. We have included various types of implanted valve devices, but determining the specific role of each valve is still necessary. Additionally, all the included studies are retrospective, which may limit our ability to control confounding variables adequately. The lack of RCTs to compare TAVI and SAVR could also affect the study’s robustness. While short-term outcomes are highly interesting, the included studies’ lack of long-term follow-up periods limits our understanding of intermediate- and long-term outcomes. Lastly, this study highlights the need for further research. The heterogeneity observed among the individual studies emphasizes the complexity and variability in outcomes associated with TAVI and SAVR. Future studies should aim to identify the factors contributing to this heterogeneity and explore additional efficacy and safety outcomes to provide a more comprehensive understanding of these interventions.

### Future recommendations

Future research directions should address the limitations of this study and further explore specific subgroups of patients. Large-scale prospective studies are needed to validate the findings and investigate the impact of evolving technologies and techniques. Further, longer-term follow-up studies with detailed efficacy and echocardiographic outcomes are warranted to confirm these results and assess potential differences in durability and valve-related complications. It would be highly important to analyze the performance of TAVI and SAVR in patients with different surgical risks to draw a definitive conclusion. Lastly, comparative cost-effectiveness analyses would also provide valuable insights for healthcare decision-makers.

## Conclusion

TAVI is a valuable option for patients with aortic regurgitation who cannot undergo SAVR. TAVI is associated with a significant reduction of in-hospital stroke, major bleeding, acute kidney injury, pneumonia, and length of hospital stay compared to SAVR.

### Supplementary Information

**Additional file 1: Supplementary Figure 1.** Forest plot of risk ratio (RR) and 95% confidence interval (CI) in the mortality with leave-one-out test. Abbreviations: TAVI; transcatheter aortic valve implantation, SAVR; aortic valve replacement, and M-H; Mantel‐Haenszel method. **Supplementary Figure 2.** Forest plot of risk ratio (RR) and 95% confidence interval (CI) in the subgroup analysis of in-hospital mortality according to the TAVI approach. Abbreviations: TAVI; transcatheter aortic valve implantation, SAVR; aortic valve replacement, and M-H; Mantel‐Haenszel method. **Supplementary Figure 3.** Forest plot of risk ratio (RR) and 95% confidence interval (CI) in the subgroup analysis of in-hospital mortality according to the country. Abbreviations: TAVI; transcatheter aortic valve implantation, SAVR; aortic valve replacement, and M-H; Mantel‐Haenszel method. **Supplementary Figure 4.** Forest plot of risk ratio (RR) and 95% confidence interval (CI) in the subgroup analysis of in-hospital stroke according to the TAVI approach. Abbreviations: TAVI; transcatheter aortic valve implantation, SAVR; aortic valve replacement, and M-H; Mantel‐Haenszel method. **Supplementary Figure 5.** Forest plot of risk ratio (RR) and 95% confidence interval (CI) in the subgroup analysis of in-hospital stroke according to the country. Abbreviations: TAVI; transcatheter aortic valve implantation, SAVR; aortic valve replacement, and M-H; Mantel‐Haenszel method. **Supplementary Figure 6.** Forest plot of risk ratio (RR) and 95% confidence interval (CI) in the subgroup analysis of acute kidney injury according to the TAVI approach. Abbreviations: TAVI; transcatheter aortic valve implantation, SAVR; aortic valve replacement, and M-H; Mantel‐Haenszel method. **Supplementary Figure 7.** Forest plot of risk ratio (RR) and 95% confidence interval (CI) in the subgroup analysis of major bleeding according to the TAVI approach. Abbreviations: TAVI; transcatheter aortic valve implantation, SAVR; aortic valve replacement, and M-H; Mantel‐Haenszel method. **Supplementary Figure 8.** Forest plot of risk ratio (RR) and 95% confidence interval (CI) in the subgroup analysis of length of hospital stay according to the TAVI approach. Abbreviations: TAVI; transcatheter aortic valve implantation, SAVR; aortic valve replacement, and IV; inverse variance method.

**Additional file 2: Supplementary Table 1.** Search strategy for each database.

**Additional file 3: Supplementary Table 2.** GRADE assessment of the certainty of the evidence.

## Data Availability

All data generated or analyzed during this study are presented in this article. On request, all additional raw data is available from the corresponding author.

## Abbreviations

AR: Aortic regurgitation
LV: Left ventricle
SAVR: Surgical aortic valve replacement
AHA: American Heart Association
ACC: American college of cardiology
ESC: European society of cardiology
EACTS: European association for cardio-thoracic surgery
TAVI: Transcatheter aortic valve implantation
FDA: Food and Drug Administration
AS: Aortic stenosis
PRISMA: Preferred reporting items for systematic reviews and meta-analyses
AMSTAR2: Assessing the methodological quality of systematic reviews 2
WOS: Web of science
CENTRAL: Cochrane library central register of controlled trials
BMI: Body mass index
NYHA: New York heart association classification of heart failure severity
AF: Atrial fibrillation
MI: Myocardial infarction
MACCE: Major adverse composite cardiac events
AKI: Acute kidney injury
PPI: Permanent pacemaker implantation
LOS: Length of hospital staying
LVEF: Left ventricular ejection fraction
LVEDD: Left ventricle end-diastolic diameter
LOS: Length of hospital staying
NOS: The Newcastle Ottawa scale
RR: Risk ratio
CI: Confidence interval
MD: Mean difference
SMD: Standardized mean difference
I^2^: Inconsistency test
GRADE: Grading of recommendations assessment, development, and evaluation
