# Supplementary material for: Transcatheter aortic valve implantation versus surgical aortic valve replacement for pure aortic regurgitation: a systematic review and meta-analysis of 33,484 patients
Source: BMC Cardiovasc Disord. 2024 Jan 23;24:65. doi: 10.1186/s12872-023-03667-0 (PMC10804466; doi:10.1186/s12872-023-03667-0)
Supplement: Supplementary file 1 — Additional file 1: Supplementary Figure 1. Forest plot of risk ratio (RR) and 95% confidence interval (CI) in the mortality with leave-one-out test. Abbreviations: TAVI; transcatheter aortic valve implantation, SAVR; aortic valve replacement, and M-H; Mantel‐Haenszel method. Supplementary Figure 2. Forest plot of risk ratio (RR) and 95% confidence interval (CI) in the subgroup analysis of in-hospital mortality according to the TAVI approach. Abbreviations: TAVI; transcatheter aortic valve implantation, SAVR; aortic valve replacement, and M-H; Mantel‐Haenszel method. Supplementary Figure 3. Forest plot of risk ratio (RR) and 95% confidence interval (CI) in the subgroup analysis of in-hospital mortality according to the country. Abbreviations: TAVI; transcatheter aortic valve implantation, SAVR; aortic valve replacement, and M-H; Mantel‐Haenszel method. Supplementary Figure 4. Forest plot of risk ratio (RR) and 95% confidence interval (CI) in the subgroup analysis of in-hospital stroke according to the TAVI approach. Abbreviations: TAVI; transcatheter aortic valve implantation, SAVR; aortic valve replacement, and M-H; Mantel‐Haenszel method. Supplementary Figure 5. Forest plot of risk ratio (RR) and 95% confidence interval (CI) in the subgroup analysis of in-hospital stroke according to the country. Abbreviations: TAVI; transcatheter aortic valve implantation, SAVR; aortic valve replacement, and M-H; Mantel‐Haenszel method. Supplementary Figure 6. Forest plot of risk ratio (RR) and 95% confidence interval (CI) in the subgroup analysis of acute kidney injury according to the TAVI approach. Abbreviations: TAVI; transcatheter aortic valve implantation, SAVR; aortic valve replacement, and M-H; Mantel‐Haenszel method. Supplementary Figure 7. Forest plot of risk ratio (RR) and 95% confidence interval (CI) in the subgroup analysis of major bleeding according to the TAVI approach. Abbreviations: TAVI; transcatheter aortic valve implantation, SAVR; aortic valve replacement, an [file 12872_2023_3667_MOESM1_ESM.docx]

**
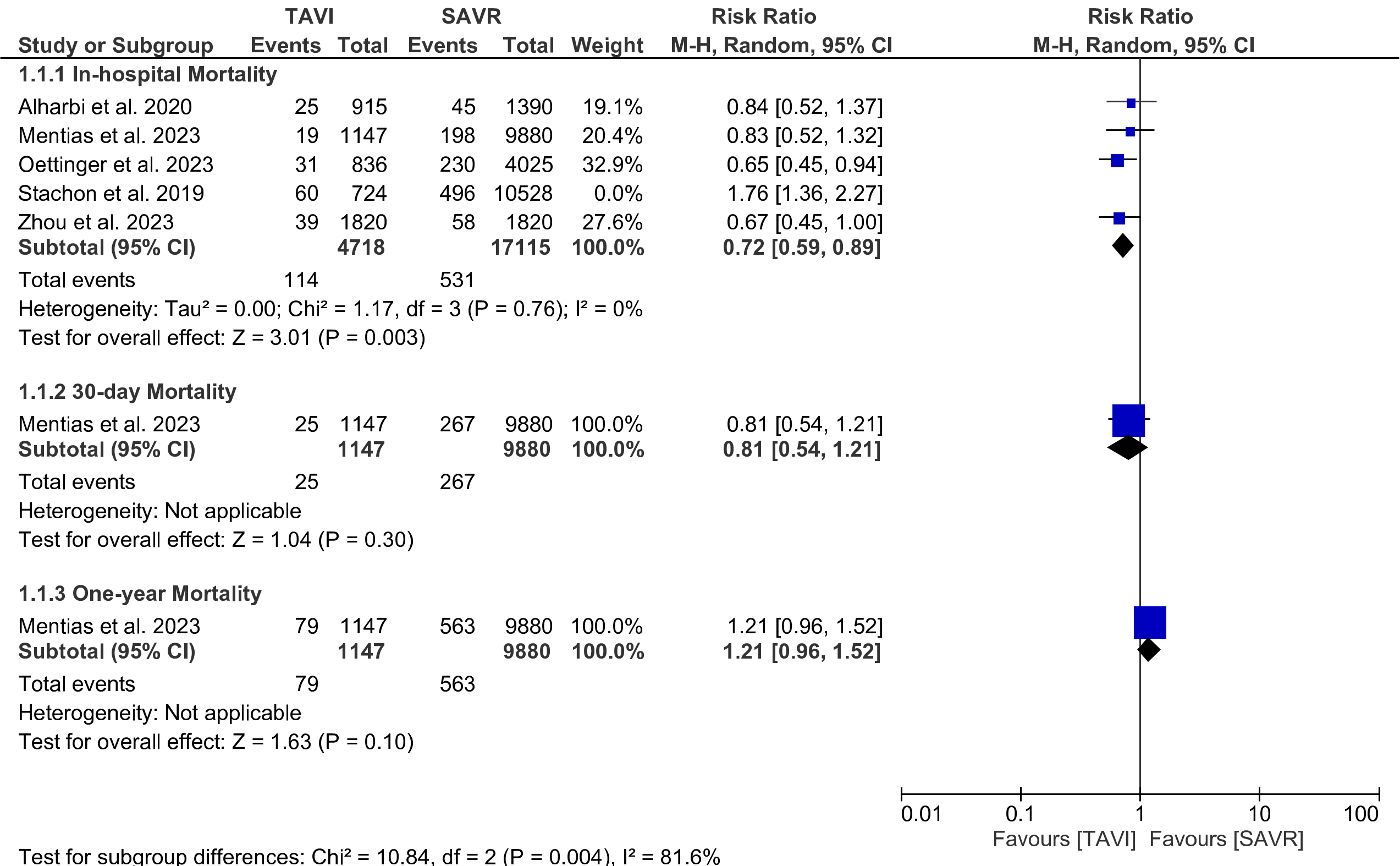
**

**Supplementary Figure 1.** Forest plot of risk ratio (RR) and 95% confidence interval (CI) in the mortality with leave-one-out test. Abbreviations: **TAVI;** transcatheter aortic valve implantation, **SAVR;** aortic valve replacement, and **M-H;** Mantel‐Haenszel method.

**
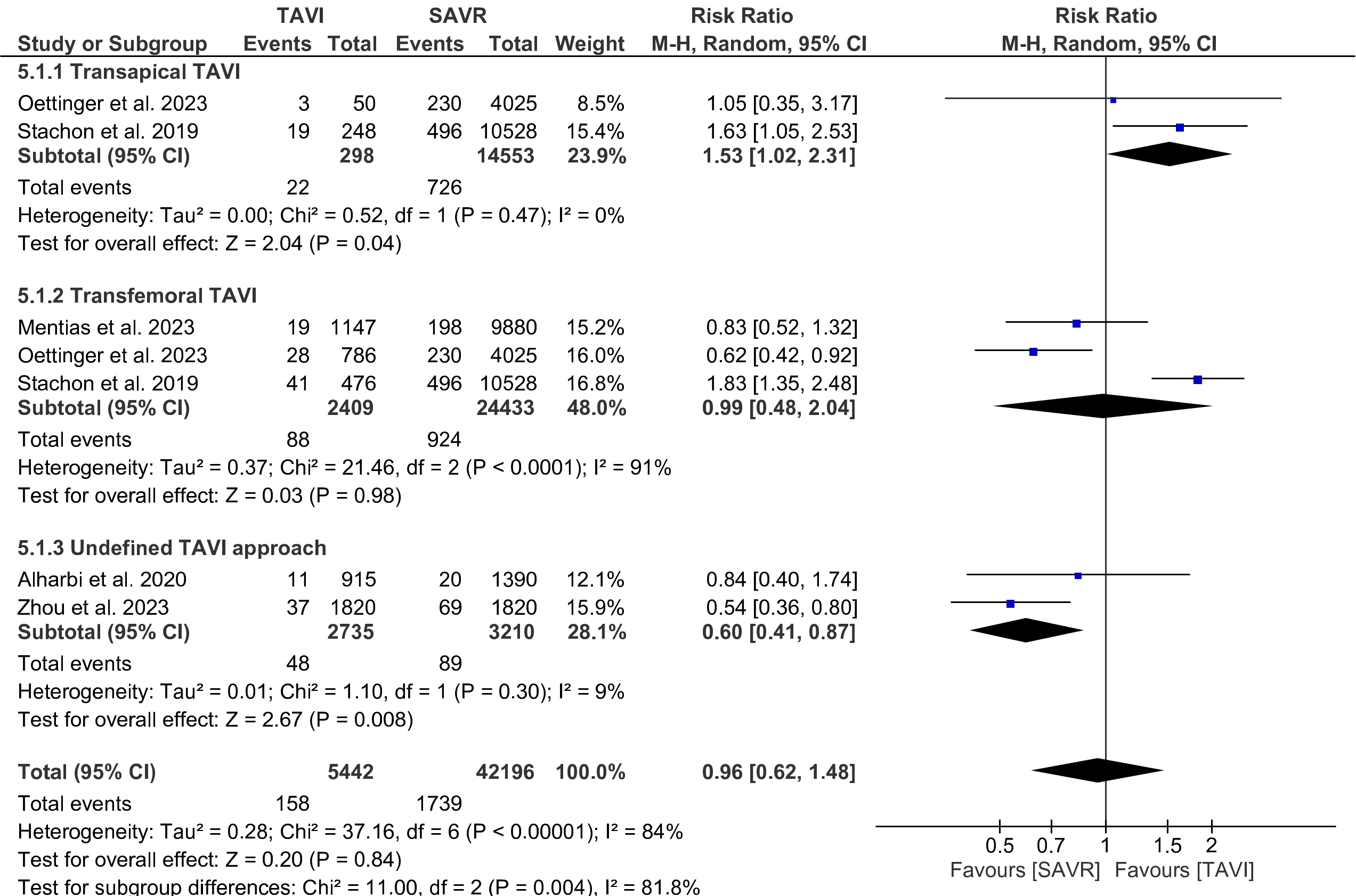
**

**Supplementary Figure 2.** Forest plot of risk ratio (RR) and 95% confidence interval (CI) in the subgroup analysis of in-hospital mortality according to the TAVI approach. Abbreviations: **TAVI;** transcatheter aortic valve implantation, **SAVR;** aortic valve replacement, and **M-H;** Mantel‐Haenszel method.


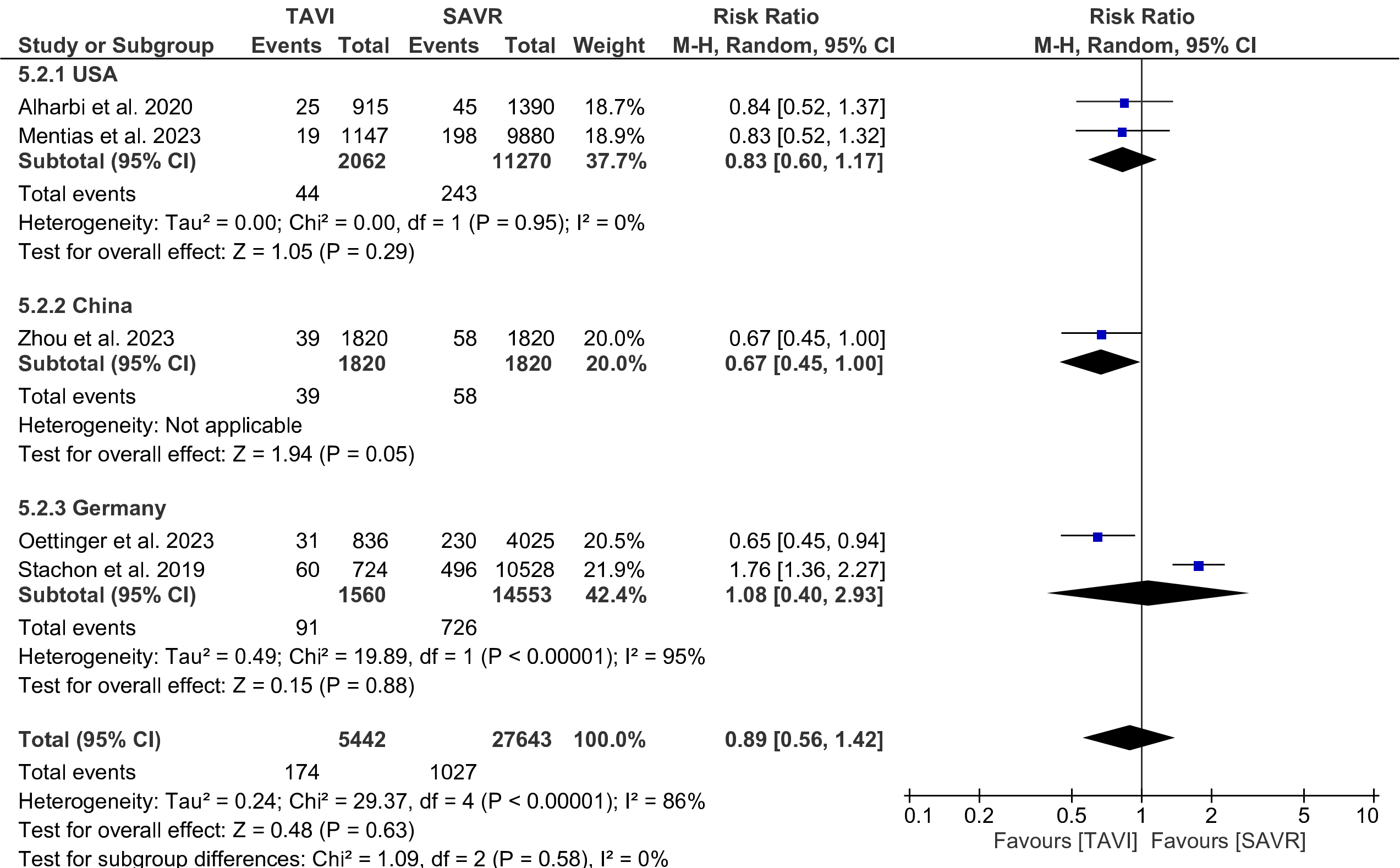


**Supplementary Figure 3.** Forest plot of risk ratio (RR) and 95% confidence interval (CI) in the subgroup analysis of in-hospital mortality according to the country. Abbreviations: **TAVI;** transcatheter aortic valve implantation, **SAVR;** aortic valve replacement, and **M-H;** Mantel‐Haenszel method.


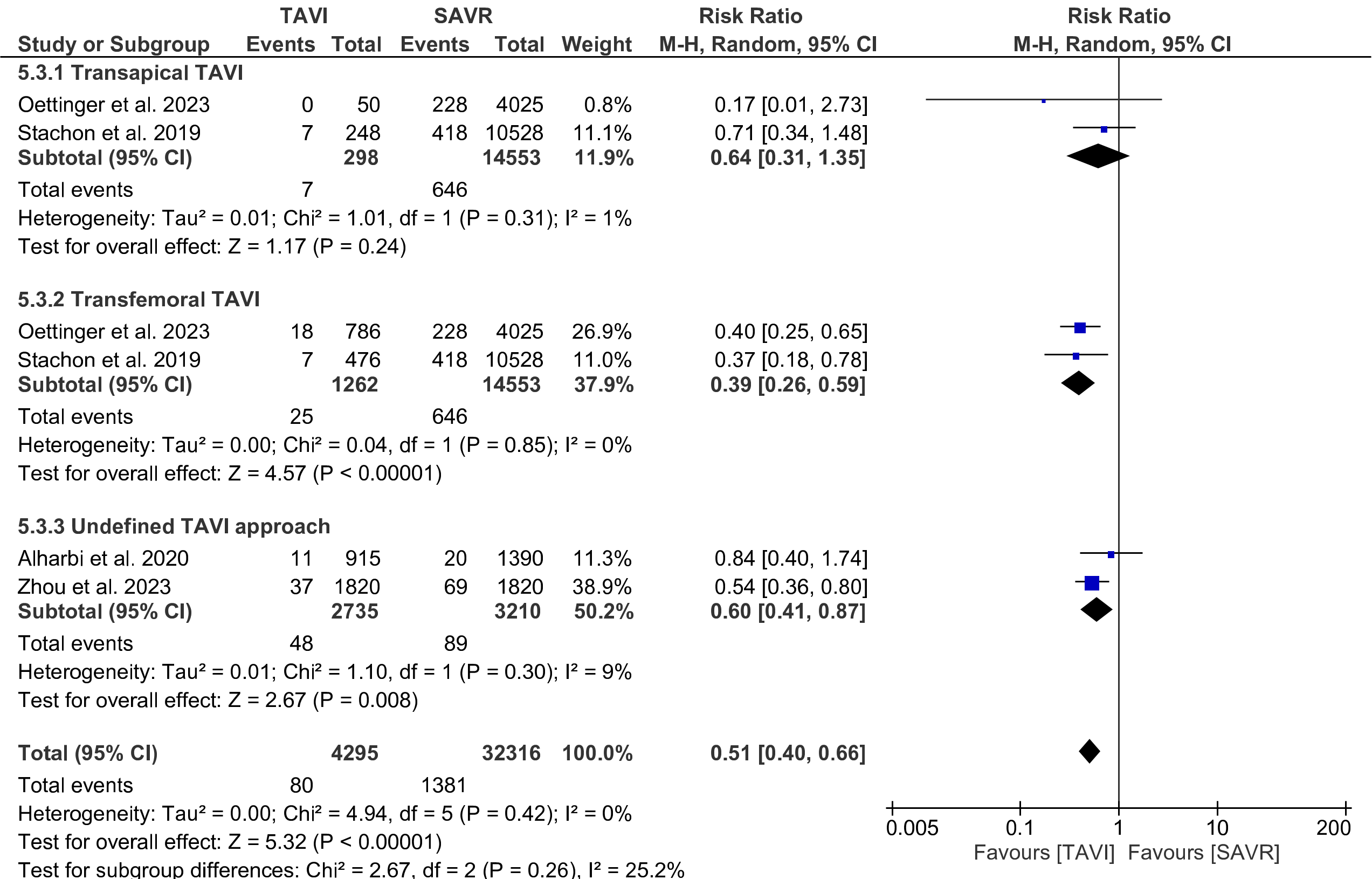


**Supplementary Figure 4.** Forest plot of risk ratio (RR) and 95% confidence interval (CI) in the subgroup analysis of in-hospital stroke according to the TAVI approach. Abbreviations: **TAVI;** transcatheter aortic valve implantation, **SAVR;** aortic valve replacement, and **M-H;** Mantel‐Haenszel method.


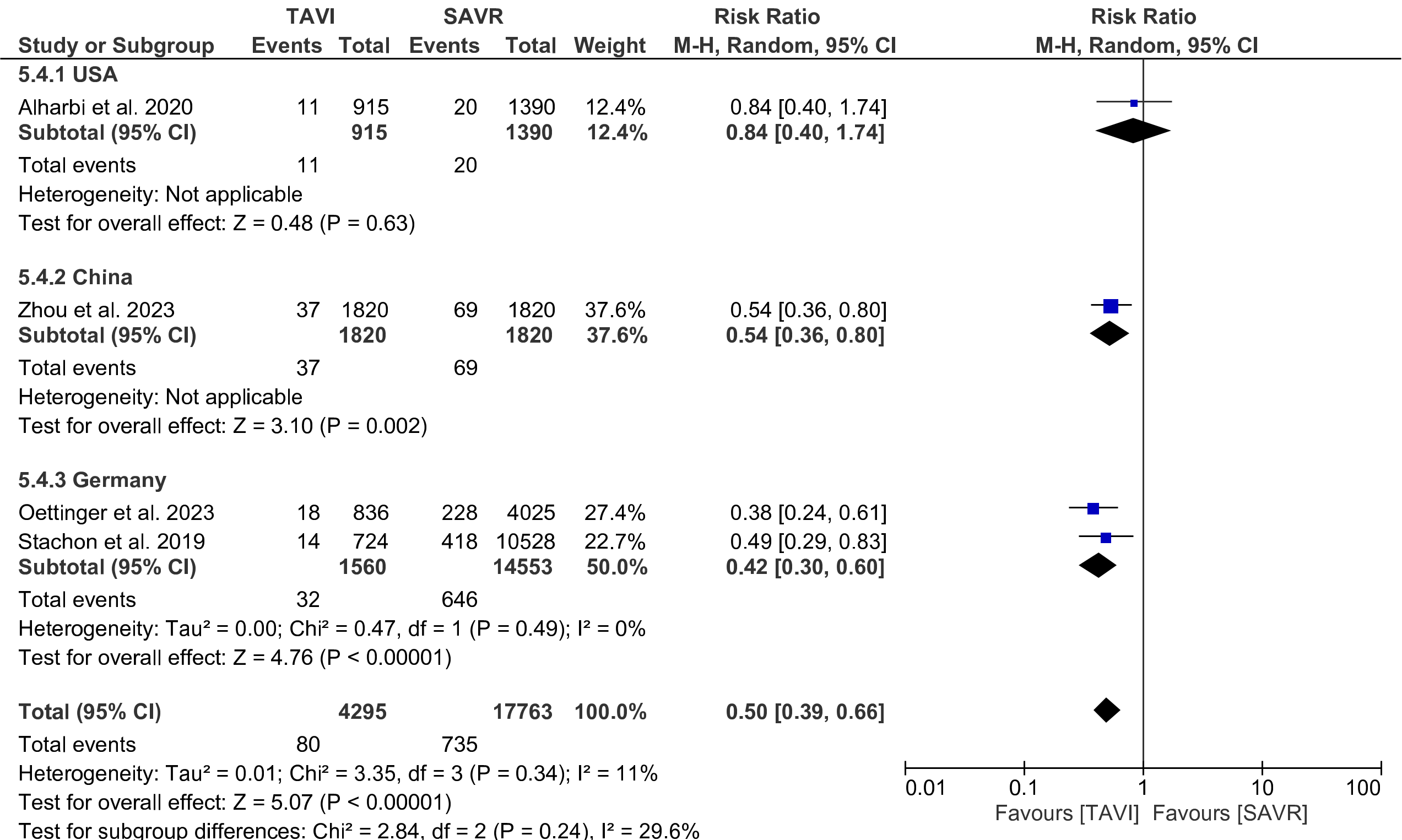


**Supplementary Figure 5.** Forest plot of risk ratio (RR) and 95% confidence interval (CI) in the subgroup analysis of in-hospital stroke according to the country. Abbreviations: **TAVI;** transcatheter aortic valve implantation, **SAVR;** aortic valve replacement, and **M-H;** Mantel‐Haenszel method.


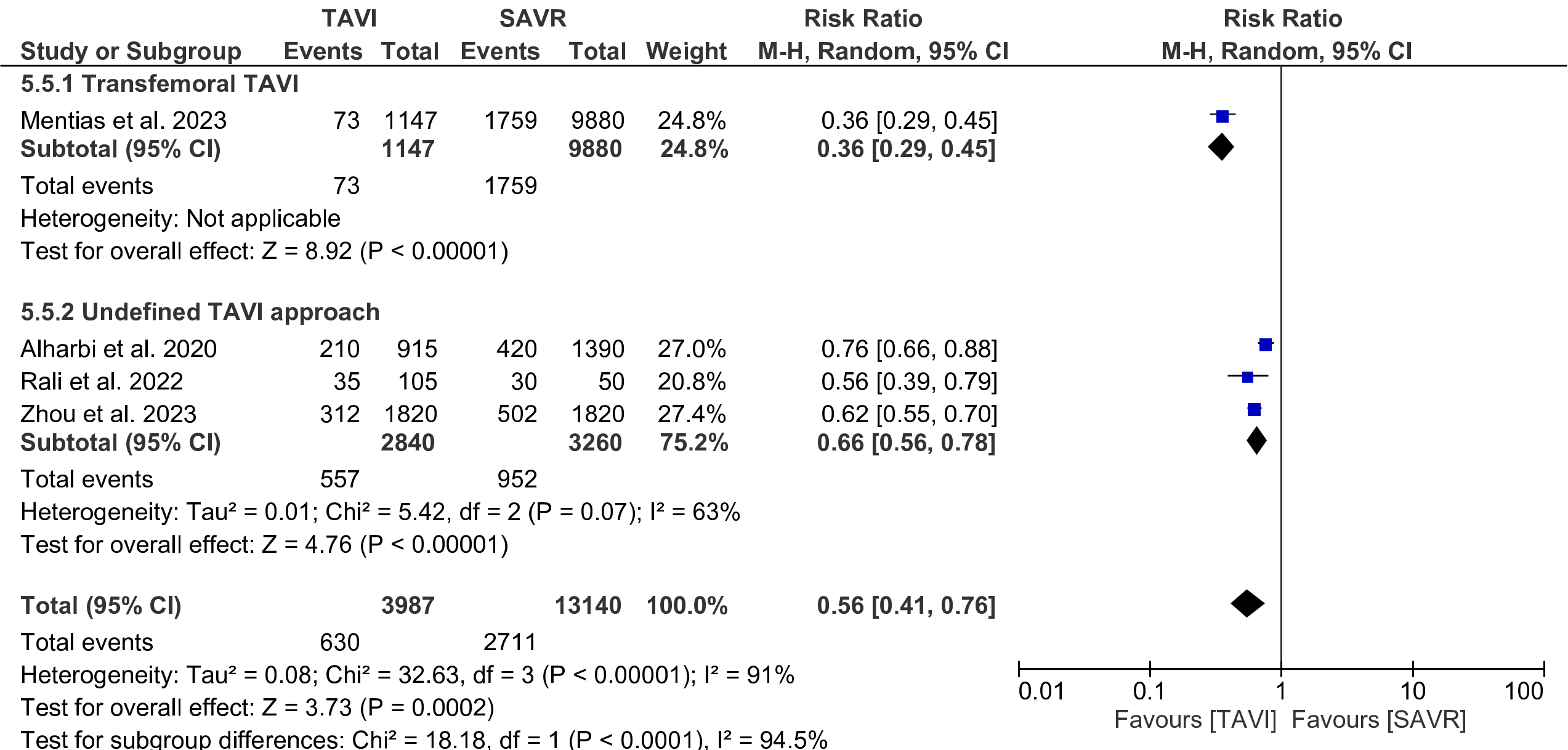


**Supplementary Figure 6.** Forest plot of risk ratio (RR) and 95% confidence interval (CI) in the subgroup analysis of acute kidney injury according to the TAVI approach. Abbreviations: **TAVI;** transcatheter aortic valve implantation, **SAVR;** aortic valve replacement, and **M-H;** Mantel‐Haenszel method.

**
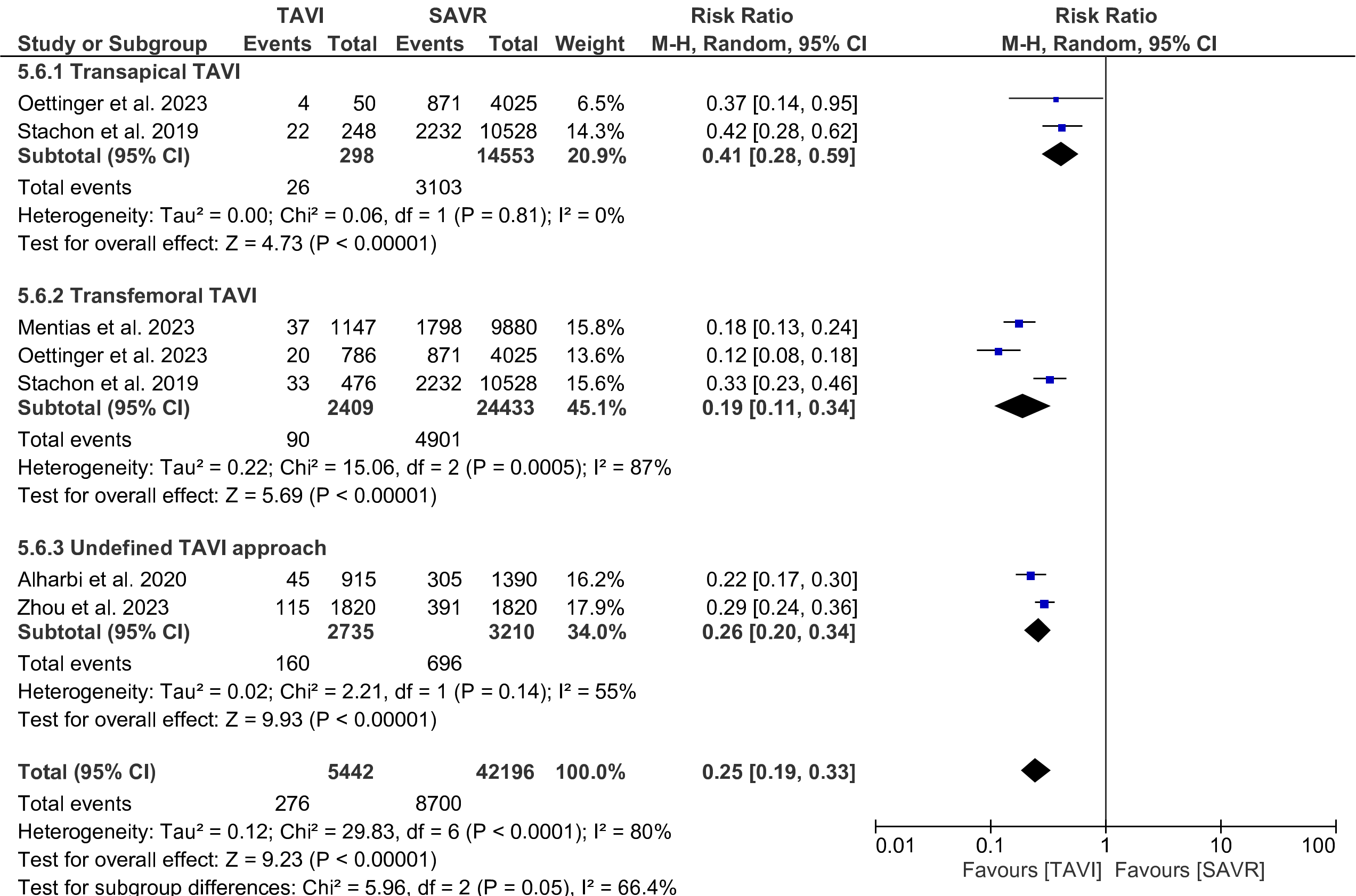
**

**Supplementary Figure 7.** Forest plot of risk ratio (RR) and 95% confidence interval (CI) in the subgroup analysis of major bleeding according to the TAVI approach. Abbreviations: **TAVI;** transcatheter aortic valve implantation, **SAVR;** aortic valve replacement, and **M-H;** Mantel‐Haenszel method.


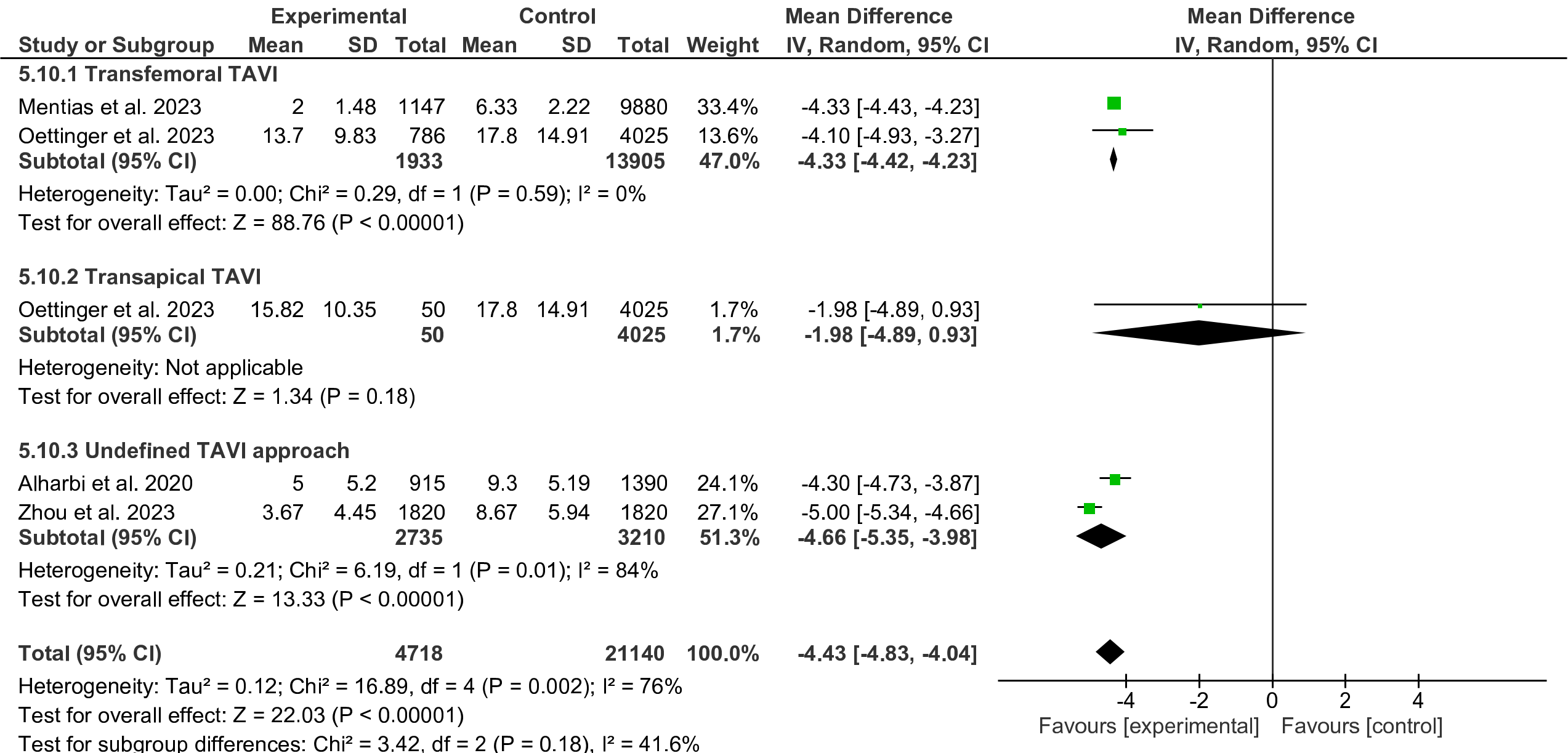


**Supplementary Figure 8.** Forest plot of risk ratio (RR) and 95% confidence interval (CI) in the subgroup analysis of length of hospital stay according to the TAVI approach. Abbreviations: **TAVI;** transcatheter aortic valve implantation, **SAVR;** aortic valve replacement, and **IV;** inverse variance method.
