# Supplementary material for: Transcatheter aortic valve implantation versus surgical aortic valve replacement for pure aortic regurgitation: a systematic review and meta-analysis of 33,484 patients
Source: BMC Cardiovasc Disord. 2024 Jan 23;24:65. doi: 10.1186/s12872-023-03667-0 (PMC10804466; doi:10.1186/s12872-023-03667-0)
Supplement: Supplementary file 2 — Additional file 2: Supplementary Table 1. Search strategy for each database. [file 12872_2023_3667_MOESM2_ESM.docx]

| Supplementary Table 1. Search strategy for each database | |
| --- | --- |
| Database | **Search strategy** |
| PubMed | ((transcatheter aortic valve implantation) OR (TAVI) OR (transcatheter aortic valve replacement) OR (TAVR)) AND ((surgical aortic valve replacement) OR (SAVR)) AND (( Aortic regurgitation) OR (Aortic insufficiency) OR (leaky aortic valve) OR (Aortic Valve Incompetence) OR (AR)) NOT (Aortic stenosis) NOT (AS) |
| Embase | ('transcatheter aortic valve implantation'/exp OR 'transcatheter aortic valve implantation' OR (transcatheter AND aortic AND ('valve'/exp OR valve) AND ('implantation'/exp OR implantation)) OR 'tavi'/exp OR tavi OR 'transcatheter aortic valve replacement'/exp OR 'transcatheter aortic valve replacement' OR (transcatheter AND aortic AND ('valve'/exp OR valve) AND ('replacement'/exp OR replacement)) OR tavr) AND ('surgical aortic valve replacement'/exp OR 'surgical aortic valve replacement' OR (surgical AND aortic AND ('valve'/exp OR valve) AND ('replacement'/exp OR replacement)) OR savr) AND ('aortic regurgitation'/exp OR 'aortic regurgitation' OR (aortic AND ('regurgitation'/exp OR regurgitation)) OR 'aortic insufficiency'/exp OR 'aortic insufficiency' OR (aortic AND insufficiency) OR 'leaky aortic valve' OR (leaky AND aortic AND ('valve'/exp OR valve)) OR 'aortic valve incompetence'/exp OR 'aortic valve incompetence' OR (aortic AND ('valve'/exp OR valve) AND incompetence) OR ar) NOT ('aortic stenosis'/exp OR 'aortic stenosis' OR (aortic AND ('stenosis'/exp OR stenosis))) NOT as |
| Scopus | TITLE-ABS-KEY ( ( ( transcatheter AND aortic AND valve AND implantation ) OR ( tavi ) OR ( transcatheter AND aortic AND valve AND replacement ) ) AND ( ( surgical AND aortic AND valve AND replacement ) OR ( savr ) ) AND ( ( aortic AND regurgitation ) OR ( aortic AND insufficiency ) OR ( leaky AND aortic AND valve ) OR ( aortic AND valve AND incompetence ) OR ( ar ) ) ) AND NOT TITLE-ABS-KEY ( ( aortic AND stenosis ) OR ( as ) ) |
| Web of Science | ((transcatheter aortic valve implantation) OR (TAVI) OR (transcatheter aortic valve replacement) OR (TAVR)) (All Fields) and ((surgical aortic valve replacement) OR (SAVR)) (All Fields) and (( Aortic regurgitation) OR (Aortic insufficiency) OR (leaking aortic valve) OR (Aortic Incompetence) OR (AR)) (All Fields) not ((Aortic stenosis)) (Topic) not (AS) (Topic) |
| Cochrane CENTRAL | ((transcatheter aortic valve implantation) OR (TAVI) OR (transcatheter aortic valve replacement) OR (TAVR)) AND ((surgical aortic valve replacement) OR (SAVR)) AND (( Aortic regurgitation) OR (Aortic insufficiency) OR (leaky aortic valve) OR (Aortic Valve Incompetence) OR (AR)) NOT (Aortic stenosis) NOT (AS) in All Text |
