# Supplementary material for: Transcatheter aortic valve implantation versus surgical aortic valve replacement for pure aortic regurgitation: a systematic review and meta-analysis of 33,484 patients
Source: BMC Cardiovasc Disord. 2024 Jan 23;24:65. doi: 10.1186/s12872-023-03667-0 (PMC10804466; doi:10.1186/s12872-023-03667-0)
Supplement: Supplementary file 3 — Additional file 3: Supplementary Table 2. GRADE assessment of the certainty of the evidence. [file 12872_2023_3667_MOESM3_ESM.docx]

| **Supplementary Table 2. GRADE assessment of the certainty of the evidence** | | | | | | | | | | | | |
| --- | --- | --- | --- | --- | --- | --- | --- | --- | --- | --- | --- | --- |
| **Certainty assessment** | | | | | | | **№ of patients** | | **Effect** | | **Certainty** | **Importance** |
| **№ of studies** | **Study design** | **Risk of bias** | **Inconsistency** | **Indirectness** | **Imprecision** | **Other considerations** | **TAVI** | **SAVR** | **Relative (95% CI)** | **Absolute (95% CI)** |  |  |
| **In-hospital mortality** | | | | | | | | | | | | |
| 5 | observational studies | not serious | serious | not serious | not serious | all plausible residual confounding would suggest spurious effect, while no effect was observed | 174/5442 (3.2%) | 1027/27643 (3.7%) | **RR 0.89** (0.56 to 1.42) | **4 fewer per 1,000** (from 16 fewer to 16 more) | ⨁⨁◯◯ Low | CRITICAL |
| **In-hospital stroke** | | | | | | | | | | | | |
| 4 | observational studies | not serious | not serious | not serious | not serious | all plausible residual confounding would suggest spurious effect, while no effect was observed | 80/4295 (1.9%) | 735/17763 (4.1%) | **RR 0.50** (0.39 to 0.66) | **21 fewer per 1,000** (from 25 fewer to 14 fewer) | ⨁⨁⨁◯ Moderate | CRITICAL |
| **Postoperative atrial fibrillation** | | | | | | | | | | | | |
| 2 | observational studies | not serious | serious | not serious | serious | all plausible residual confounding would suggest spurious effect, while no effect was observed | 436/2062 (21.1%) | 3681/11270 (32.7%) | **RR 0.26** (0.02 to 3.80) | **242 fewer per 1,000** (from 320 fewer to 915 more) | ⨁◯◯◯ Very low | IMPORTANT |
| **In-hospital acute kidney injury** | | | | | | | | | | | | |
| 4 | observational studies | not serious | serious | not serious | not serious | all plausible residual confounding would suggest spurious effect, while no effect was observed | 630/3987 (15.8%) | 2711/13140 (20.6%) | **RR 0.56** (0.41 to 0.76) | **91 fewer per 1,000** (from 122 fewer to 50 fewer) | ⨁⨁◯◯ Low | IMPORTANT |
| **Major bleeding** | | | | | | | | | | | | |
| 5 | observational studies | not serious | serious | not serious | not serious | all plausible residual confounding would suggest spurious effect, while no effect was observed | 276/5442 (5.1%) | 5597/27643 (20.2%) | **RR 0.23** (0.17 to 0.32) | **156 fewer per 1,000** (from 168 fewer to 138 fewer) | ⨁⨁◯◯ Low | IMPORTANT |
| **permanent pacemaker implantation** | | | | | | | | | | | | |
| 2 | observational studies | not serious | not serious | not serious | not serious | none | 507/3882 (13.1%) | 945/13090 (7.2%) | **RR 1.68** (1.50 to 1.88) | **49 more per 1,000** (from 36 more to 64 more) | ⨁⨁◯◯ Low | IMPORTANT |
|  | | | | | | | | | | | | |
| **Delirium** | | | | | | | | | | | | |
| 2 | observational studies | not serious | serious | not serious | serious | all plausible residual confounding would suggest spurious effect, while no effect was observed | 100/1560 (6.4%) | 1216/14553 (8.4%) | **RR 0.68** (0.25 to 1.88) | **27 fewer per 1,000** (from 63 fewer to 74 more) | ⨁◯◯◯ Very low | IMPORTANT |
| **Pneumonia** | | | | | | | | | | | | |
| 2 | observational studies | not serious | not serious | not serious | not serious | all plausible residual confounding would suggest spurious effect, while no effect was observed | 74/2735 (2.7%) | 161/3210 (5.0%) | **RR 0.53** (0.40 to 0.70) | **24 fewer per 1,000** (from 30 fewer to 15 fewer) | ⨁⨁⨁◯ Moderate | IMPORTANT |
| **Sepsis** | | | | | | | | | | | | |
| 2 | observational studies | not serious | serious | not serious | serious | none | 42/2735 (1.5%) | 127/3210 (4.0%) | **RR 0.15** (0.01 to 2.23) | **34 fewer per 1,000** (from 39 fewer to 49 more) | ⨁◯◯◯ Very low | IMPORTANT |
| **Length of hospital stay** | | | | | | | | | | | | |
| 4 | observational studies | not serious | serious | not serious | not serious | strong association all plausible residual confounding would suggest spurious effect, while no effect was observed | 4718 | 17115 | - | MD **4.76 day fewer** (5.27 fewer to 4.25 fewer) | ⨁⨁⨁◯ Moderate | IMPORTANT |

**CI:** confidence interval; **MD:** mean difference; **RR:** risk ratio
